# Photoelectrocatalytic H_2_ evolution from integrated photocatalysts adsorbed on NiO†
†Electronic supplementary information (ESI) available: Electrochemical and photoelectrochemical characterisation, surface analysis, TA and TRIR spectra. See DOI: 10.1039/c8sc02575d


**DOI:** 10.1039/c8sc02575d

**Published:** 2018-10-04

**Authors:** Nils Põldme, Laura O'Reilly, Ian Fletcher, Jose Portoles, Igor V. Sazanovich, Michael Towrie, Conor Long, Johannes G. Vos, Mary T. Pryce, Elizabeth A. Gibson

**Affiliations:** a School of Natural and Environmental Science , Newcastle University , Newcastle upon Tyne , NE1 7RU , UK . Email: Elizabeth.gibson@ncl.ac.uk; b School of Chemical Sciences , Dublin City University , Dublin 9 , Ireland . Email: Mary.pryce@dcu.ie; c NEXUS XPS Laboratory , Newcastle University , Stephenson Building , Newcastle upon Tyne , NE1 7RU , UK . Email: nexus@ncl.ac.uk; d Central Laser Facility , Research Complex at Harwell , STFC Rutherford Appleton Laboratory , Harwell Campus , Didcot , Oxfordshire OX11 0QX , UK . Email: Igor.Sazanovich@stfc.ac.uk

## Abstract

A new approach to increasing the faradaic efficiency of dye-sensitised photocathodes for H_2_ evolution from water, using integrated photocatalysts, furnished with ester groups on the peripheral ligands, [Ru(decb)_2_(bpt)PdCl(H_2_O)](PF_6_)_2_ (**1**) and [Ru(decb)_2_(2,5-bpp)PtI(CH_3_CN)](PF_6_)_2_ (**2**), (decb = 4,4′-diethylcarboxy-2,2′-bipyridine, bpp = 2,2':5′,2′′-terpyridine, bpt = 3,5-bis(2-pyridyl)-1,2,4-triazole) is described. Overall, **1**|NiO is superior to previously reported photocathodes, producing photocurrent densities of 30–35 μA cm^–2^ at an applied bias of –0.2 V *vs.* Ag/AgCl over 1 hour of continuous white light irradiation, resulting in the generation of 0.41 μmol h^–1^ cm^–2^ of H_2_ with faradaic efficiencies of up to 90%. Furthermore, surface analysis of the photocathodes before and after photoelectrocatalysis revealed that the ruthenium bipyridyl chromophore and Pd catalytic centre (**1**) were photochemically stable, highlighting the benefits of the approach towards robust, hybrid solar-to-fuel devices.

## Abstract 2

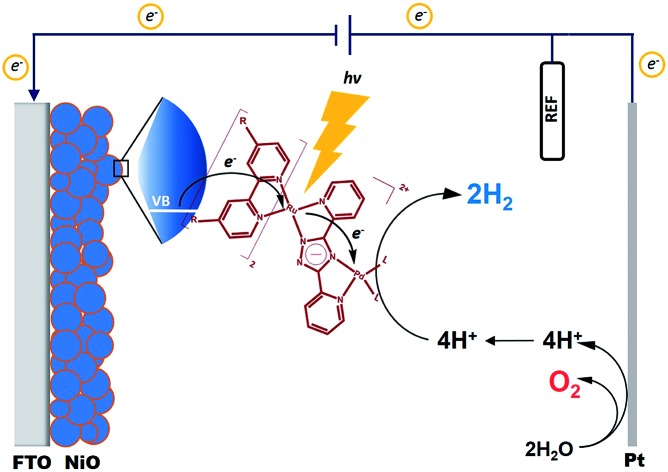
A new approach to increasing the faradaic efficiency of dye-sensitised photocathodes for H_2_ evolution from water is described, using integrated photocatalysts based on a ruthenium 4,4′-diethoxycarboxy-2,2′-bipyridine chromophore linked *via* terpyridine or triazole to a Pd or Pt-based H^+^ reduction catalyst.

## Introduction

In recent years, p-type dye sensitised solar cells (p-DSCs), have attracted considerable attention. This is because of their potential applications in photoelectrocatalytic devices for H_2_ evolution, CO_2_ reduction and also in the advancement of tandem DSCs.^1^,^2^ Frequently, NiO-based electrodes are used in these devices. The efficiency of conventional DSCs can be increased, in theory, by replacing the Pt counter electrode with a photocathode which captures light transmitted by a TiO_2_-based photoanode. In addition, these devices can be augmented with catalysts to drive photoelectrocatalytic water splitting in so-called dye sensitised photoelectrochemical cells (DSPEC).

DSPECs have many important advantages over other water splitting systems. Firstly, in contrast to homogeneous systems sacrificial electron donors are no longer required. This is because the electrons for H^+^ reduction are provided by water oxidation at the anode and delivered to the photocathode through the external circuit.^3^,^4^ Secondly, by combining the molecular photosensitiser with the molecular or colloidal catalyst on a semiconductor surface, the photon absorption, charge transfer, and catalyst activity can be optimised, leading to higher efficiency and lower processing costs.^5^ Thirdly, using a molecular photosensitiser provides the opportunity to tune the absorption properties of the system. Combining a dye-sensitised photocathode with a dye-sensitised photoanode, presents an exciting opportunity to use the low energy part of the visible spectrum on one electrode and the high energy part on the other.^5^ Thus, more of the spectrum is harnessed, resulting in these tandem cells achieving a high photon to H_2_ efficiency.

Mesoporous NiO has been used in p-DSCs and tandem cells since the late 1990's. It is one of the few stable p-type semiconductors with a wide band-gap and, consequently, the electrode film does not compete with the dye for visible light absorption.^6^–^11^ Recently, it has been used in a DSPEC to reduce H^+^ to H_2_.^5^,^12^–^17^ Nanostructured NiO films can be produced at reasonably low temperatures in a cost-efficient manner, making them ideal for large-scale applications. NiO is also stable in the mildly acidic aqueous environments required for H_2_ production. The valence band (VB) is approximately 0.4 V *vs.* NHE in pH 6.8 phosphate buffer (approx. 0.62 V *vs.* NHE at pH 3), which lies between the frontier orbitals of typical photosensitisers, such as Ru(bpy)_3_^2+^.^18^ Excitation of a photosensitiser adsorbed on NiO can result in extraction of an electron from the NiO (hole injection) reducing the sensitiser and initiating the photocatalytic reaction.^18^,^19^ The exact mechanism for H_2_ evolution, which requires two electrons, is unclear at this stage.^17^,^20^–^22^

Unfortunately, rapid and efficient recombination of the reduced dye and the hole in the NiO valence band reduces photocatalytic efficiency of these systems. Obviously, research into ways to increase the photosensitiser efficiency and reduce the non-productive back reaction will be of enormous benefit.^23^,^24^ Understanding the excited state dynamics of the sensitiser is essential, while accurate modelling of the long-lived charge separated state and the localisation of the charge remote from the NiO surface can help in reducing the efficiency of the charge recombination processes.

**Fig. 1:**
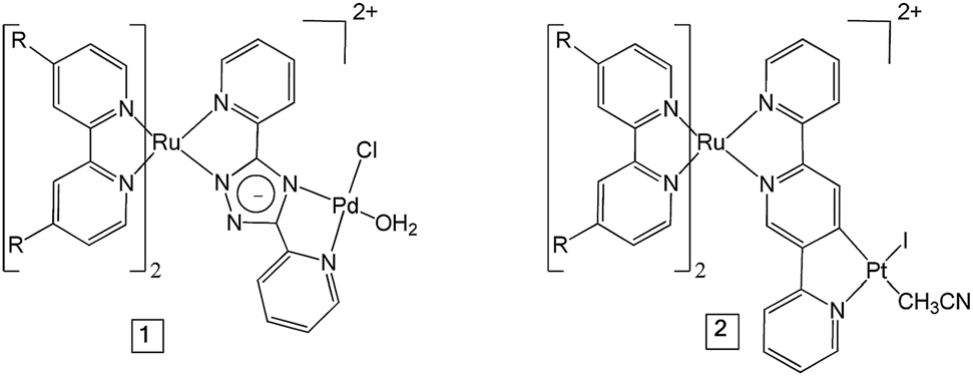
The structures of the dye-catalyst assemblies **1** and **2** (R = CO_2_Et).

As mentioned above, polypyridyl ruthenium(ii) complexes have been explored as photosensitisers. We have previously studied the H_2_ generating capability of compound **2** (Fig. 1) in solution (CH_3_CN/TEA/H_2_O), and obtained a turnover number (TON) of 650 after 6 h irradiation at 470 nm.^25^ Analysis of transient absorption (TA) data indicated that the ester ligands on bipyridine lowers the energy of the ^3^MLCT (metal-to-ligand charge transfer) state in which the unpaired spin is located on a peripheral carboxy-bipy ligand. This excited state is long-lived compared to the unsubstituted bipyridine complex perhaps explaining the high TONs achieved for this complex. In this manuscript, we have used time-resolved infrared (TRIR) spectroscopy and time resolved UV-visible spectroscopy (TA) to characterise the excited states, and the excited state dynamics of complexes **1** and **2** (Fig. 1) in CD_3_CN and also when immobilised on NiO surfaces (**1**|NiO and **2**|NiO), respectively. H_2_ production by **1**|NiO and **2**|NiO in DSPEC's was confirmed under two different applied potentials, *E*_appl_ = –0.4 V and –0.2 V *vs.* Ag/AgCl, thereby negating the need for sacrificial agents. The performance and stability of the photocathodes are discussed below.

## Results

### Photocatalyst adsorption

**Fig. 2:**
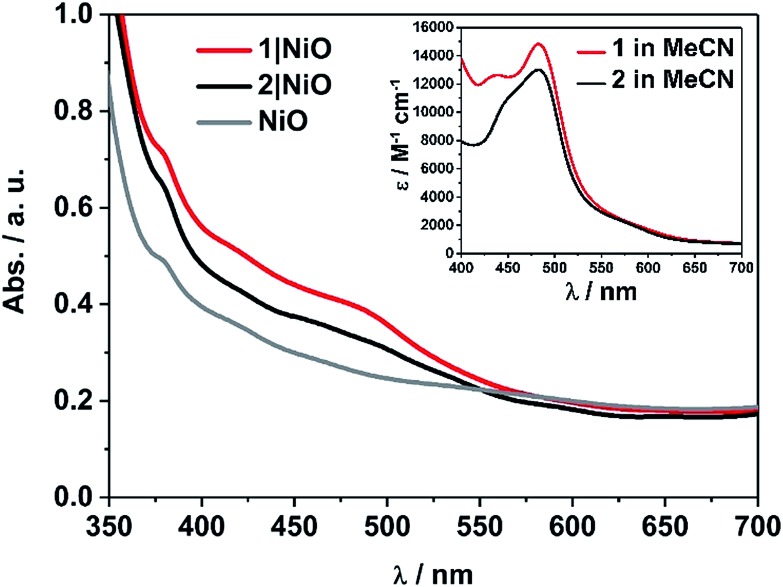
UV-vis absorption spectra of **1** (red) and **2** (black) adsorbed on NiO and bare NiO (gray). Inset: UV-vis absorption spectra of **1** (red) and **2** (black) in MeCN (0.028 mM and 0.032 mM, respectively).

The UV-visible absorption spectra of complexes **1** and **2** dissolved in acetonitrile solution and immobilised on NiO are provided in Fig. 2. The spectra are generally broader and blue-shifted when compared to homogeneous solution (*λ*_max_ = 490 nm for **1** and 480 nm for **1**|NiO, *λ*_max_ = 480 nm for **2** and *ca.* 470 nm for **2**|NiO) which is consistent with an electronic interaction between the ground state of the dye and the NiO surface. Time-Dependent Density Functional calculations (TD-DFT), described below, suggest that a Ru-to-carboxy-bipy charge-transfer transition is responsible for the low-energy absorption maximum.

The loading of the photocatalyst onto the NiO surface was quantified from the absorption of **1** and **2** on NiO (Fig. 2).^74^ For **1**, assuming that the absorption coefficient does not change substantially on grafting, the photocatalyst loading was 9 nmol cm^–2^, and for **2**, it was 5.3 nmol cm^–2^. These values are reasonably similar, consistent with the similar anchoring system, and are a similar order of magnitude to dye-sensitised photocathodes reported elsewhere.^26^ The FTIR spectra (Fig. S37 and S38†) of complexes **1** and **2** in KBr have a carbonyl band at 1724 and 1726 cm^–1^, respectively, and when the complexes are immobilised on NiO, the carbonyl bands shift to 1720 cm^–1^ for both complexes. In both cases there is a marginal shift to lower frequency, which could indicate an interaction between the ester and NiO.

### Time resolved IR and TA spectroscopy

**Fig. 3:**
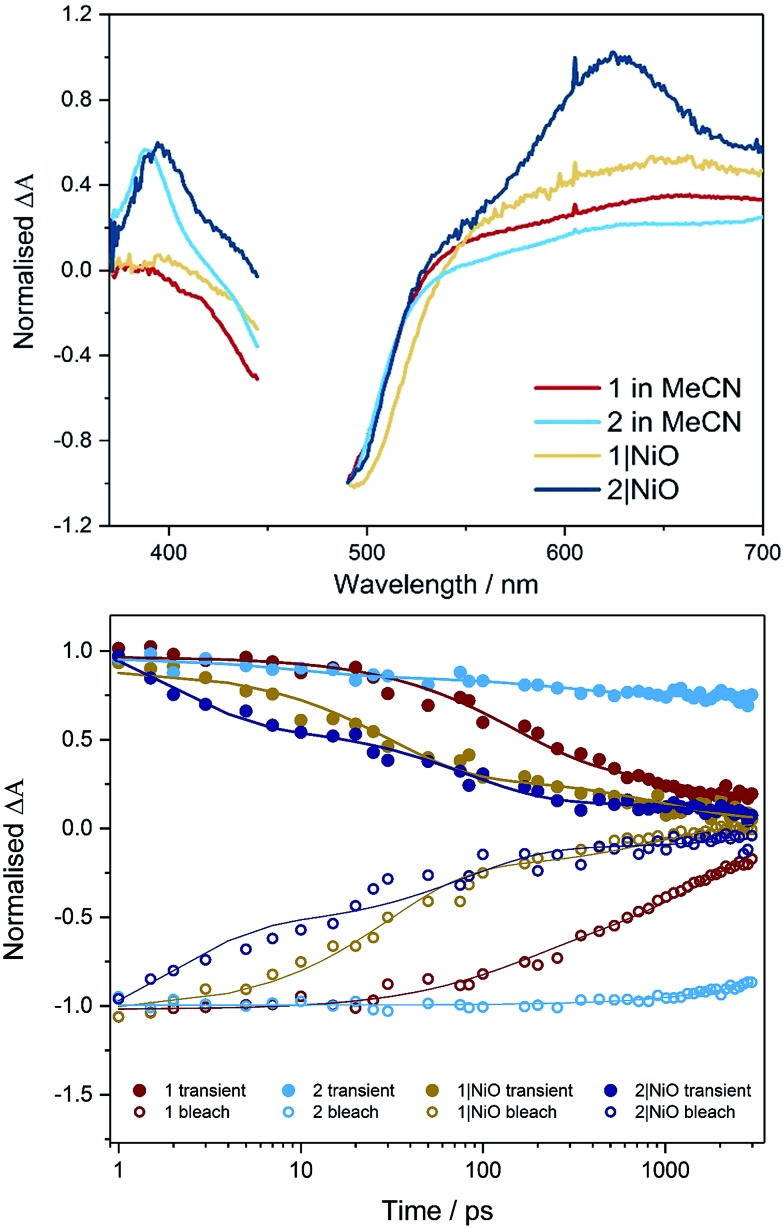
Transient absorption spectra (top, 1 ps) and kinetic traces (bottom) for **1** (red) and **2** (light blue) in CD_3_CN and **1**|NiO (yellow) and **2**|NiO (dark blue) following excitation (*λ* = 470 nm) probed at 490 nm (bleach, open symbols) and 620 nm (transient, filled symbols). On the bottom figure, the rings represent data points and the lines are the exponential fit to the data.

**Fig. 4:**
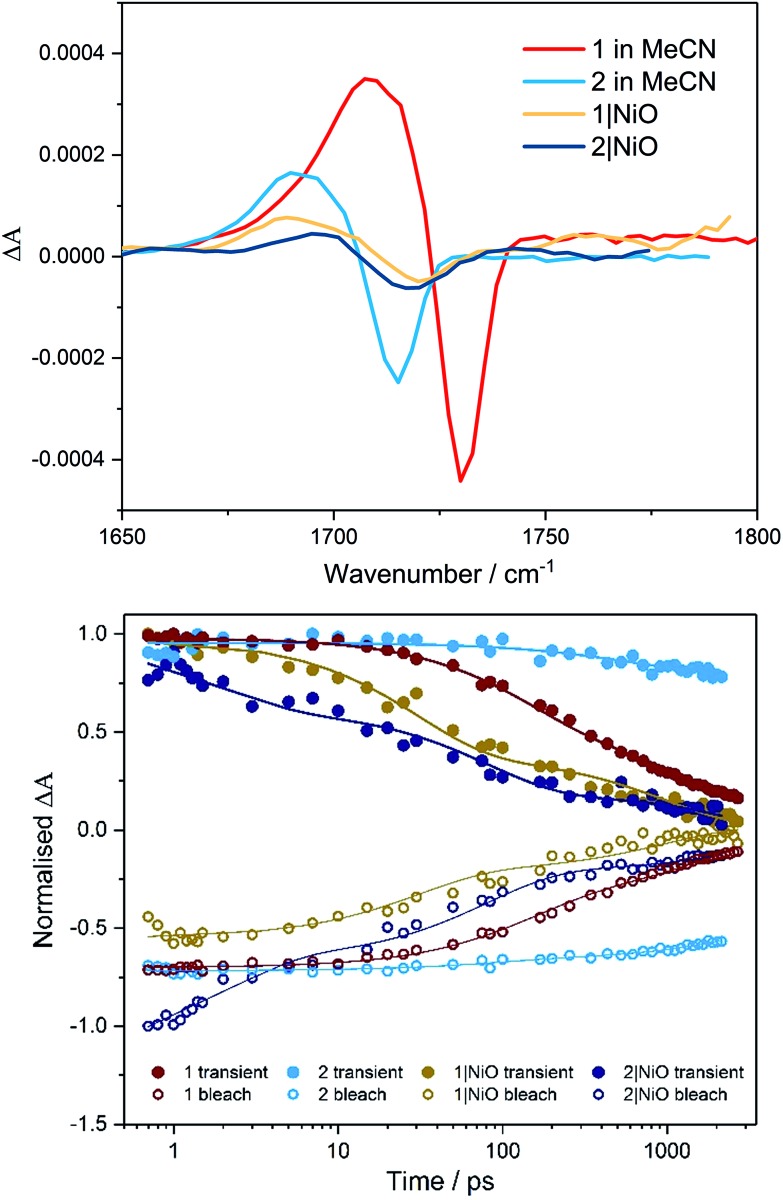
TRIR Spectra (top, 1 ps) and kinetic traces (bottom) for **1** (red) and **2** (light blue) in CD_3_CN and **1**|NiO (yellow) and **2**|NiO (dark blue) following excitation (*λ* = 470 nm). On the bottom figure, the symbols represent data points (open = bleach, filled = transient absorption) and the lines are the exponential fit to the data.

The photoelectrochemical process is initiated when a dye absorbed on the NiO surface absorbs light, stimulating hole injection into the NiO material and reduction of the dye.^19^ To probe the photoexcitation, hole injection and recombination dynamics, time-resolved infrared and transient absorption studies were performed on both complex **1** and **2** in deuterated acetonitrile solution and when immobilised onto NiO films, following excitation at 470 nm. The data are shown in Fig. 3 and 4 below and Fig. S27–S33 in the ESI.†

### Transient absorption spectroscopy in solution

The transient absorption spectra obtained following pulsed photolysis (*λ*_exc_ = 470 nm) of complexes **1** and **2**, in CD_3_CN solution are given in Fig. 3 and S27–S29 in the ESI.† In the case of compound **2**, a ground state bleach occurs within the pulse, a broad absorption that extends from 500 nm is evident, together with an additional absorption band at *ca.* 380 nm, which also persists beyond the 3 ns window of the experiment. These transient absorption features (LMCT and π → π* transition on bpy˙^–^), are typical for ruthenium bipyridyl complexes and are assigned to the ^3^MLCT excited state species which persist on the ns to μs timescale.^25^,^27^ For compound **1**, only two features are evident, a ground state bleach which occurs within the pulse, and a broad absorption extending from *ca.* 560 nm. This broad absorption feature decays over approximately *τ* = 2 ns, concomitant with recovery of the ground state bleach. On closer inspection using global fitting of the transient absorption, unlike **2**, two time constants were extracted for **1** (*τ*_1_ = 137, *τ*_2_ = 1830 ps), which were associated with a 40 nm red-shift the visible band. Furthermore, TD-DFT calculations on the model complex, [Ru(dmcb)_2_(bpt)PdCl(H_2_O)](PF_6_)_2_ suggest that the initially formed singlet Ru-to-dmcb charge-transfer excited state rapidly crosses to the triplet surface forming a triplet Ru-to-bpt charge transfer state and not a Ru-to-dmcb charge-transfer state. This explains the absence of absorptions *ca.* 380 nm in TA spectra for complex **1**.

### Transient absorption on NiO

When compounds **1** and **2** were immobilised onto NiO films (**1**|NiO, **2**|NiO) (Fig. 3, S27, S28, S30 and S31†), the excited species were generated within the laser pulse (200 fs). The initial spectral shape is consistent with the ^3^MLCT excited species superimposed on a second coexisting species which is assigned to the reduced dye and is consistent with hole injection into the NiO (holes injected into NiO have a broad featureless absorption spectrum extending throughout the red-NIR region^18^). To extract the dynamics of these processes, global analysis was performed and the fit was evaluated by inspecting the systematic residuals. Fitting a parallel multiexponential model to the data gave the decay associated difference spectra shown in Fig. S30 and S31 in the ESI.† For **1**|NiO, two lifetimes were extracted, *τ*_1_ ≈ 30 ps, *τ*_2_ ≈ 1 ns. The shape of the long-lived species, *τ*_2_, is similar to the excited state absorption spectra of **1** in solution. The short-lived species, *τ*_1_, absorbs broadly between 540 to 700 nm, with a maximum at 648 nm and does not contain the positive band at 385 nm, which is characteristic of the reduced decb ligand.^28^ We assign *τ*_1_ to the reduced dye, **1**^–^|NiO^+^, and *τ*_2_ to the triplet ^3^MLCT excited species, 1*|NiO.

In the case of **2**|NiO, global fitting gave three distinguishable components (Fig. 3), *τ*_1_ ≈ 2 ps, *τ*_2_ ≈ 80 ps, *τ*_3_ ≈ 4 ns. All three contained a positive transient at 370–400 nm, which is associated with the reduced decb ligand, the ground state bleach between 440 to 540 nm (both of these spectral characteristics were evident in the TA experiments carried out in solution as described above), but differed in the region beyond 520 nm. The spectrum associated with the nanosecond component, *τ*_3_, is similar in shape to the excited state absorption spectra of **2** in solution. The shortest-lived component, *τ*_1_, contained a Gaussian-shaped absorption band at *ca.* 630 nm. On this timescale (<5 ps), there is approximately 40% recovery of the parent depletion at 500 nm. This species is attributed to hole injection and the formation of the reduced dye (**2**^–^|NiO^+^), and the timebases are similar to that previously reported for bis[2,20-bipyridine][4,4′-dicarboxy-2,20-bipyridine]ruthenium(ii) sensitised NiO.^29^ The component in between, *τ*_2_, contains a broad excited state absorption signal above 520 nm which is similar in structure to ligand-to-metal charge transfer (LMCT) transitions associated with the terpyridyl bridging ligand.^28^ The presence of this component, which is absent in the solution spectra, suggests that the equilibrium between excited states is different when the dye is adsorbed on NiO compared to solution.

### Time-resolved IR in solution

TRIR studies in the fingerprint region (Fig. 4, S32 and S33†) were performed in deuterated acetonitrile. Following excitation at 470 nm, a depletion of the carbonyl group was observed at 1730 cm^–1^ for compound **1** and at 1714 cm^–1^ for compound **2**. A new band to the low energy side of the parent bleach was detected for both complexes and this is assigned to the carbonyl band of the ^3^MLCT excited state species. For complex **1**, this band decays with concomitant recovery of the parent bleach over *τ* = *ca.* 2 ns. In the case of compound **2**, both the excited state feature and the parent bleach persist for longer than 2 ns. The shorter lifetime for compound **1** containing the triazole bridging ligand, agrees with studies previously observed for ruthenium triazole complexes.^30^

### Time-resolved IR on NiO

Time-resolved IR studies were also performed using **1**|NiO and **2**|NiO (Fig. 4, S32 and S33 in ESI†). As observed in the solution studies, upon excitation, depletion of the carbonyl group occurs within the laser pulse (200 fs) for both compounds, with a new carbonyl band at lower frequency as previously observed in solution studies (discussed above). In the case of **1**|NiO, near full recovery of the parent depletion and decay of the band at 1689 cm^–1^ is observed within 200 ps. Consistent with the solution studies, these spectral changes are assigned to the ^3^MLCT excited state species. In addition, there is a further species with very weak bands at higher frequency to that of the parent depletion for both **1**|NiO and **2**|NiO. As these bands are very weak and at the edge of the detection window, it is difficult to get reliable kinetic data. However, similarly to the ^3^MLCT excited species which forms within the excitation pulse, these weak bands in the range 1750–1810 cm^–1^, also form within this timeframe. For **2**|NiO, similar spectral features are observed to that for complex **1**.

### Computational studies of [Ru(bipy)_2_(bpt)PdCl](PF_6_)_2_, [Ru(dmcb)_2_(bpt)PdCl](PF_6_)_2_, [Ru(dmcb)_2_(bpt)PdCl(H_2_O)](PF_6_)_2_ and [Ru(dmcb)_2_(bpt)PdCl](PF_6_)

Following the satisfactory optimisation of the ground-state singlet structure for the three model complexes at the B3LYP/LanL2DZ level, the TD-DFT method was used to estimate the energy and electronic structure of the fifty low-lying singlet excited states. These calculations were undertaken firstly to verify that the methods used reliably represented the onset absorptions of the complexes when compared to the experimental UV-visible spectra. Secondly, these calculations can be used to construct electron density difference maps for each of the excited states, and these maps are useful in characterising the nature of the excited state in terms of electron drift relative to the ground-state electronic structure. For instance, MLCT transitions can be clearly characterised, as in the case of the lowest energy optically accessible singlet state (S_17_, 432.6 nm) of [Ru(bipy)_2_(bpt)PdCl](PF_6_)_2_ modelled in acetonitrile (Fig. S21(b)†). Here this excited state can be characterised as a ruthenium to bpt charge-transfer state. In contrast however, the lowest energy optically accessible singlet state of [Ru(dmcb)_2_(bpt)PdCl](PF_6_)_2_ (S_12_, 479.3 nm) has mainly ruthenium to dmcb charge transfer character (Fig. S26†). Clearly the ester-substituents on the “ancilliary” bipy ligands have a dramatic effect on the nature of the accessible excited states and have a greater role in the catalytic mechanism than as simple binding sites to the NiO surface.

**Fig. 5:**
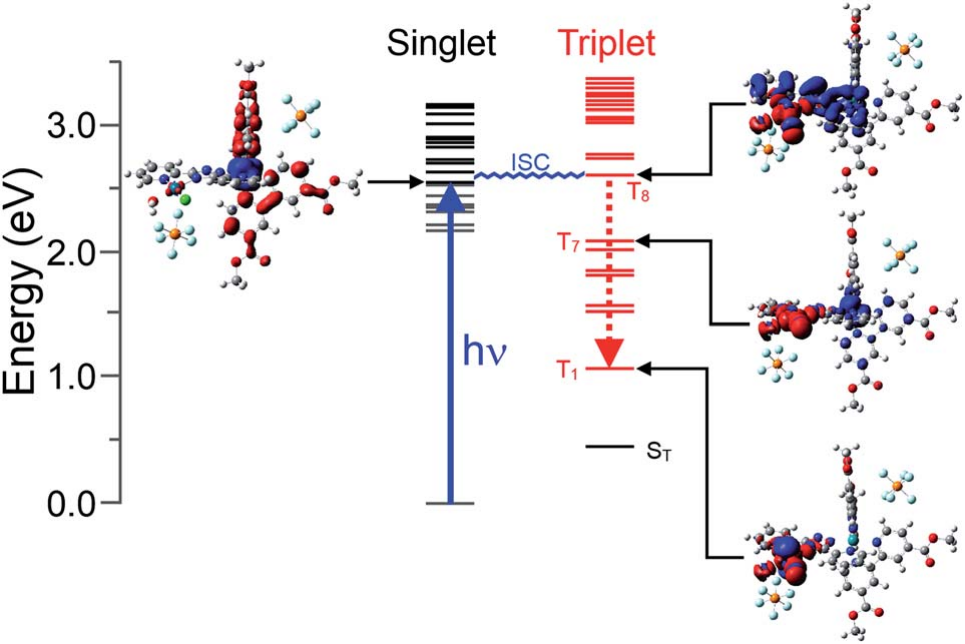
A photophysical model for the excitation of [Ru(dmcb)_2_(bpt)PdCl(H_2_O)](PF_6_)_2_ into its lowest energy optically accessible singlet excited state (8^th^ excited state, corresponding to a photon energy of 484 nm in water, black lines) followed by inter system crossing (ISC) to the triplet surface (red lines) and internal conversion to the triplet surface; the singlet state energy at the triplet geometry is indicated by S_T_. Vertical excitation to the lowest energy optically accessible state is indicated as the blue arrow, and the electron density difference maps or selected states are also presented to either side of the energy level.

As the catalytic processes take place in aqueous environments, the electronic structure of [Ru(dmcb)_2_(bpt)PdCl(H_2_O)](PF_6_)_2_ in water was modelled both on the singlet and triplet surfaces. The lowest energy optically accessible singlet (Fig. 5) excited state exhibits mainly ruthenium-to-dmcb charge-transfer character, and if it is assumed that this state is efficiently populated, then the closest triplet state in energy terms is the T_8_ which has predominantly ruthenium/bpt to palladium charge-transfer character. Thus, the intersystem crossing process results in a significant change to the electronic structure, increasing the electron density on the palladium atom and reducing the electron density on the ruthenium and bpt ligand. This explains why the TA experiments on **1** fail to produce features consistent with a Ru-to-bipy charge-transfer excited state at *ca.* 380 nm. More importantly, the energy of T_8_ lies 2.64 eV (254 kJ mol^–1^) above the ground state singlet energy which is sufficient to split water. Finally the electronic structure of the singly reduced [Ru(dmcb)_2_(bpt)PdCl](PF_6_) was modelled. The spin distribution in this doublet species was mapped, which indicated the presence of the excess spin on the Pd atom and its coordination sphere.

### Photoelectrochemistry and hydrogen evolution

**Fig. 6:**
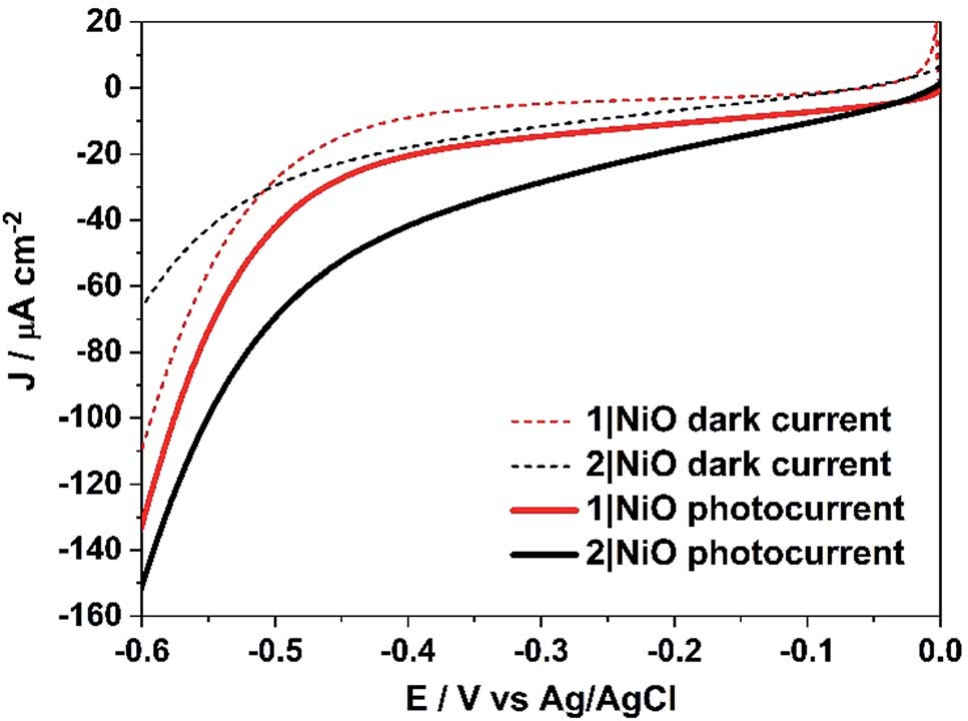
Linear sweep voltammograms of **1**|NiO and **2**|NiO immersed in aqueous 0.1 M KCl electrolyte (pH 3). The potential was swept from *E*_appl_ = 0 to –0.6 V *vs.* Ag/AgCl (3.0 M NaCl) in the dark and under simulated light illumination (AM1.5, 100 mW cm^–2^).

Linear sweep voltammetry (LSV) measurements were carried out on the **1**|NiO and **2**|NiO electrodes, immersed in aqueous electrolyte solution with 0.1 M KCl. The pH was adjusted with the addition of diluted HCl (pH 1 to pH 7). The potential was swept from *E*_appl_ = 0 V to –0.6 V *vs.* Ag/AgCl for each pH value, under chopped light conditions, during which the samples were irradiated with white light (AM1.5, 100 mW cm^–2^) over 10 s intervals, under steady-state illumination and in the dark (Fig. S2 in the ESI†). The photocurrent density increased slightly when the pH was raised. This is possibly due to slower charge injection and increased recombination at lower pH.^31^ The least acidic environment where H_2_ was detected was pH 3 and this was chosen for further experiments. At pH 3, for both **1**|NiO and **2**|NiO, the photocurrent increased compared to the dark current until *E*_appl_ = –0.4 V *vs.* Ag/AgCl (Fig. 6). At more negative potentials, the magnitude of both the dark current and photocurrent density increased considerably.

Three potentials were chosen at which to monitor H_2_ evolution, *E*_appl_ = –0.40 V *vs.* Ag/AgCl (*ca.* –0.01 V *vs.* RHE), where the photocurrent and dark current were most stable, *E*_appl_ = –0.60 V *vs.* Ag/AgCl (*ca.* –0.21 V *vs.* RHE), and *E*_appl_ = –0.20 V *vs.* Ag/AgCl (*ca.* 0.21 V *vs.* RHE), all of which are lower than the conduction band edge of TiO_2_ (*i.e.* in a tandem PEC device, the bias which will be applied by the photoanode driving the overall water splitting), approximately –0.54 V *vs.* RHE.^12^,^32^

*E*_appl_ was fixed while the current was measured and Ar was continuously flowed through the electrolyte solution and the exhaust was sampled by in-line GC analysis (see Experimental). Control experiments were carried out on a bare fluorine-doped tin oxide (FTO) substrate and a non-sensitised NiO|FTO electrode under the same conditions (pH 3 aqueous electrolyte with 0.1 M potassium hydrogen phthalate) to distinguish between the activity of the substrate and the sensitised electrode and to check for any electrochemically active impurities (see Fig. S4 in the ESI†). No H_2_ or photocurrent was detected during the control measurements with FTO and the current density was considerably lower for the bare electrodes compared to the sensitised electrodes measured under same conditions. However, although no H_2_ was detected, a steady increase in photocurrent was observed for the NiO|FTO sample at *E*_appl_ = –0.6 V. The reason for the increase in photocurrent is possibly a reduction of some Ni^3+^ surface states during illumination with white light.^33^

**Fig. 7:**
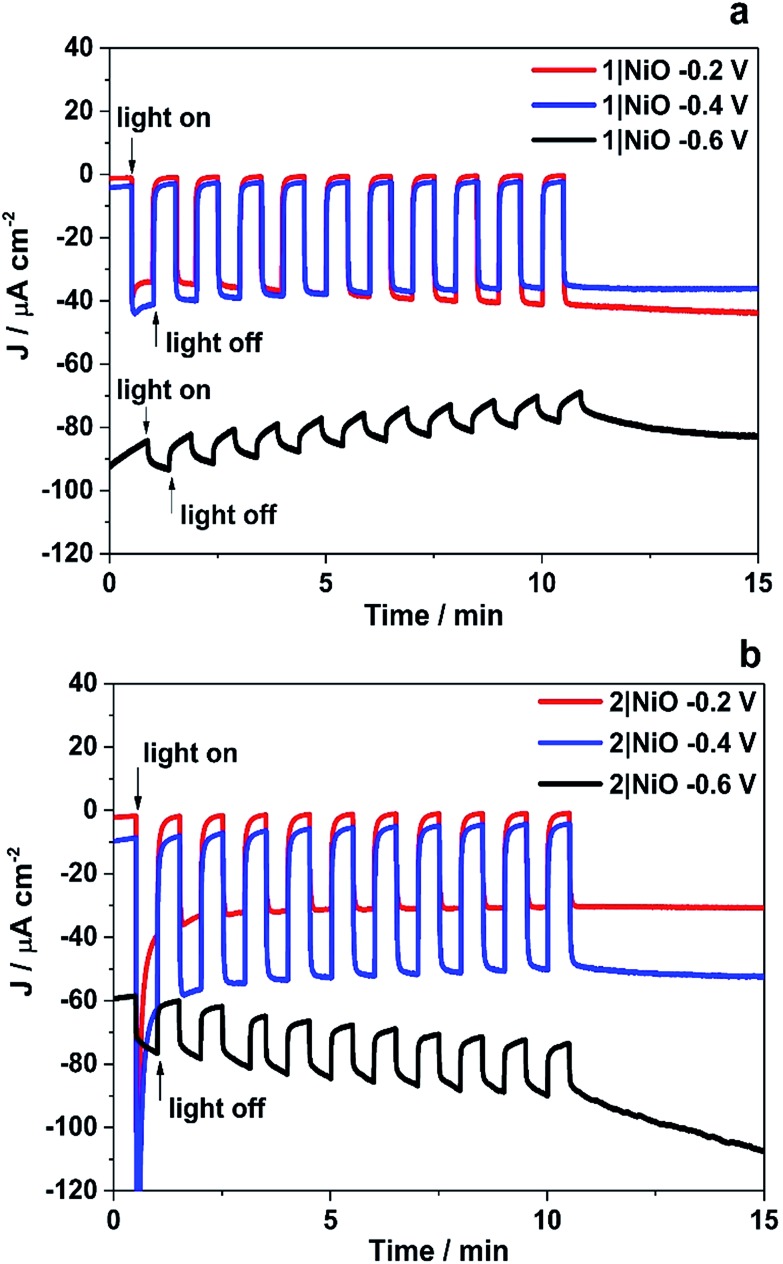
Chronoamperometry measurements of **1**|NiO (a) and **2**|NiO (b) immersed in pH 3 buffer containing 0.1 M potassium hydrogen phthalate. *E*_appl_ = –0.2 V, –0.4 V and –0.6 V *vs.* Ag/AgCl (3.0 M NaCl). Chopped light illumination was applied with 30 s intervals (10 cycles of dark current/photocurrent).

The trends observed for the photoelectrodes **1**|NiO and **2**|NiO during the chronoamperometry experiments under chopped light irradiation (Fig. 7) were consistent with the LSV experiments (Fig. 6), which is representative of the good reproducibility of the system. For **1**|NiO, stable cathodic photocurrents were recorded at *E*_appl_ = –0.2 V (*J*_photo_ = *ca.* 37 μA cm^–2^) and *E*_appl_ = –0.4 V (*J*_photo_ = *ca.* 44 μA cm^–2^), over 10 dark/light cycles (Fig. 7(a)). The dark current recorded during those measurements was negligible compared to the photocurrent and only increased slightly when the more negative potential was applied. For **2**|NiO, a significant increase in photocurrent was observed at the more negative bias, *E*_appl_ = –0.4 V (*J*_photo_ = *ca.* 53 μA cm^–2^) compared to *E*_appl_ = –0.2 V (*J*_photo_ = *ca.* 31 μA cm^–2^) (Fig. 7(b)). As for **1**|NiO, the dark current was very low compared to the photocurrent. An initial spike in the photocurrent was observed during the chronoamperometry of **2**|NiO at *E*_appl_ = –0.2 V and –0.4 V. We attribute this rapidly decaying photocurrent to local capacitance effects, the reduction of the dye-catalyst assembly and/or Ni^3+^ on the surface.^17^,^21^ Another explanation could be slow electron transfer from the catalyst to the H^+^ or slow diffusion of the products from the pores leading to charge recombination.^15^,^17^,^22^ The spike in photocurrent was much less pronounced for **1**|NiO which indicates that charge-transfer in the forward direction (*e.g.* from NiO to the catalyst, to the substrate) is faster than charge-recombination.^17^,^20^–^22^ After these initial spikes, the current was stable and the absence of capacitive features after the first on–off cycle suggests that the current is not diffusion limited. The chopped light illumination was followed by a constant white light illumination for up to a 1 h to test the electrode stability (Fig. S3 ESI†). A small, steady decrease in photocurrent was observed for both samples under almost all *E*_appl_, due to the decrease in active surface area upon bubble formation on the electrode. This behaviour was also observed for a Pt-coated FTO electrode (Fig. S4 in the ESI†) and on shaking the cell, the current was restored. To remove the formed bubbles on the sample surface, the cell was manually shaken in the end of each measurement and additional samples of outlet gas were analysed with GC until no more H_2_ was detected. Unlike the photocurrent, the dark current at *E*_appl_ = –0.2 V and *E*_appl_ = –0.4 V did not vary over the duration of the experiment and the magnitude was <10% of the total current recorded. At more negative potentials than *E*_appl_ = –0.6 V, more significant changes in current *vs.* time were observed. A larger dark current (*J*_dark_ = 78 μA cm^–2^) was recorded for **1**|NiO (Fig. 7a) and **2**|NiO (Fig. 7b), compared to the previous measurements, which decreased over time. In addition, for **2**|NiO, the dark current decreased when the light was switched off and the photocurrent increased when light was turned on. This is an indication of changes occurring on the electrode surface during irradiation.

**Fig. 8:**
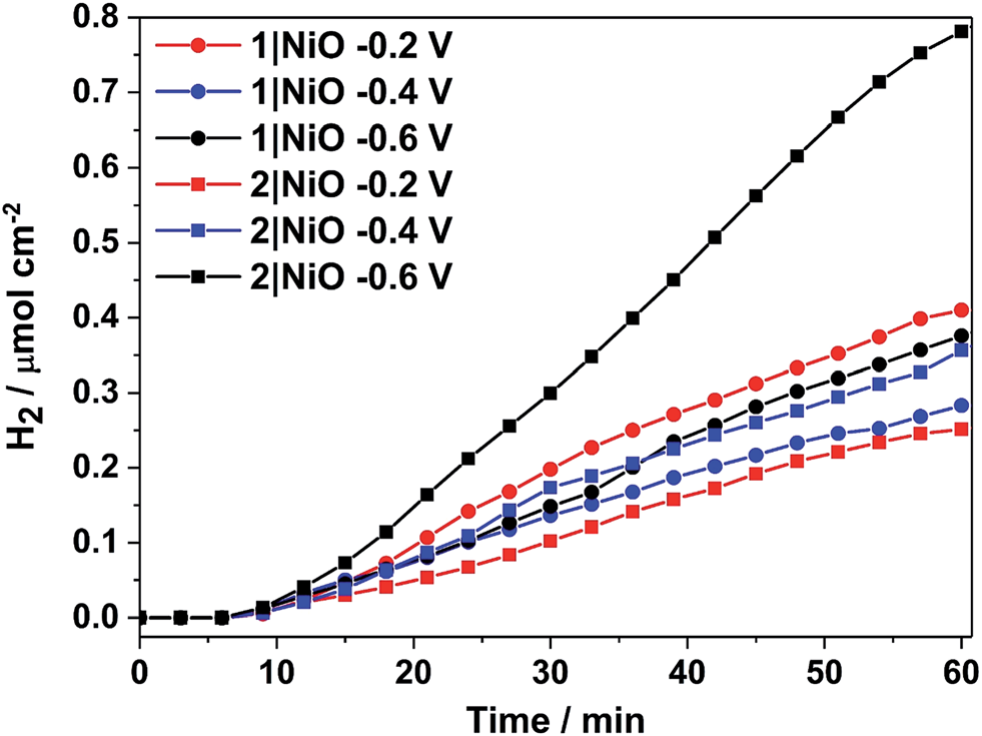
Cumulative photoelectrocatalytic H_2_ production by **1**|NiO and **2**|NiO during the chronoamperometry measurements at different *E*_appl_ (Fig. 7 and S3 in the ESI†). Chopped light illumination was applied during the first 10 min followed by constant light illumination until the end of the measurement (AM1.5, 100 mW cm^–2^).

H_2_ was detected from both **1**|NiO and **2**|NiO under white light illumination at *E*_appl_ = –0.2 V to –0.6 V and the volume detected *vs.* time is presented in Fig. 8. No H_2_ was detected during the control measurements in the dark, except for **2**|NiO under *E*_appl_ = –0.6 V *vs.* Ag/AgCl, where the rate of H2 evolution decreased after the light was switched off. The faradaic efficiencies (*η*_Far_) calculated for **1**|NiO and **2**|NiO are presented in Table 1. The efficiencies of both photocathodes decrease with the increase in applied potential. Reported values of *η*_Far_ for H_2_ evolution elsewhere range from <10% to >100%.^2^ Our values are estimates as the peaks in the chromatogram corresponding to H_2_ were small and integrated manually. The photocurrent density and volume of H_2_ were affected by differences in film thickness and care was taken to ensure these were consistent between samples (1.5 μm). Turnover numbers (TONs) for the immobilised photocatalysts **1**|NiO and **2**|NiO were estimated from the number of dye molecules adsorbed and the quantity of H_2_ produced at each *E*_appl_ during 1 hour and are included in Table 1. The best TON for **1** resulted from measurement under *E*_appl_ = –0.2 V *vs.* Ag/AgCl, which was 46 and increasing the potential resulted in lower TON. The estimated TONs for **2**|NiO were higher than **1**|NiO at all *E*_appl_. However, as the photocatalyst was shown to be decomposing to Pt^0^ during the post-catalysis characterisation of electrodes by XPS (Fig. 10), the TON for **2** is not accurate.

**Fig. 9:**
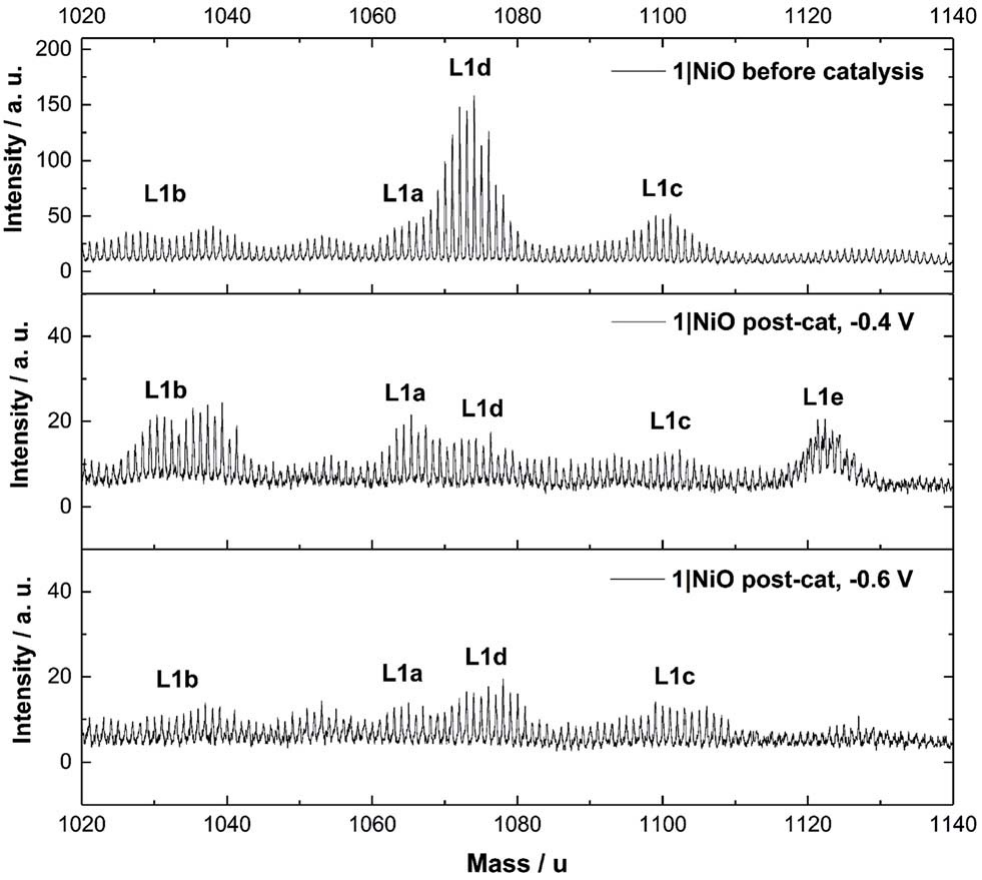
ToF-SIMS positive ion spectra of 1|NiO taken from three different samples: top: – pre-catalysis, middle: post-catalysis at –0.4 V *vs.* Ag/AgCl and bottom: post-catalysis –0.6 V *vs.* Ag/AgCl. 1020–1140 mass per unit range. Assignments are as follows: L1a – [Ru(decb)_2_(bpt)PdCl]^2+^ calcd. *m* = 1065, L1b – [Ru(decb)_2_(bpt)Pd]^3+^ calcd. *m* = 1030, L1c – [Ru(decb)_2_(bpt)PdCl(H_2_O)_2_]^2+^ calcd. *m* = 1101, L1d – [Ru(decb)_2_(bpt)Pd(CH_3_CN)]^3+^ calcd. *m* = 1071, L1e – [Ru(decb)_2_(bpt)PdCl(H_2_O)_3_]^2+^ calcd. *m* = 1119.

**Table 1:** Photocurrent, faradaic efficiencies and H_2_ production during photoelectrocatalysis of **1**|NiO and **2**|NiO under AM1.5 illumination (100 mW cm^–2^)^*a*^

	**1**|NiO			**2**|NiO		
*E*_appl_/V *vs.* Ag/AgCl	–0.2	–0.4	–0.6	–0.2	–0.4	–0.6
*J*_photo_/μA cm^–2^	34.8 ± 1.4	31.2 ± 8.7	12.3 ± 1.9	31.8 ± 6.2	47.6 ± 7.5	43.7 ± 27.1
*J*_total_/μA cm^–2^	35.6 ± 1.8	34.2 ± 9.4	88.2 ± 4.9	33.2 ± 7.1	52.5 ± 12.0	114.7 ± 37.6
*P*_photo_/%	97.8	91.2	14	95.8	90.7	38.1
*η*_Far_/%	88.6	67.5	32.3*	59.1	56.9	44.4*
[H_2_]/μmol h^–1^ cm^–2^	0.41	0.28	0.37	0.25	0.36	0.78
TON	46	31	41	47	68	147

^*a*^ *J*_photo_ is the average photocurrent, *J*_total_ is the photocurrent plus dark current, *P*_photo_ is the percentage of photocurrent of the total current and *η*_Far_ is the faradaic efficiency of H_2_ production. *η*_Far_ was calculated using *J*_photo_ in case of *E*_appl_ = –0.2 V and *E*_appl_ = –0.4 V. *η_Far_ was calculated from the overall charge passed during the measurement (dark and photocurrent) in case of *E*_appl_ = –0.6 V *vs.* Ag/AgCl. TON calculated from the dye-loading (mol cm^–2^) and the [H_2_] over 1 hour.

**Fig. 10:**
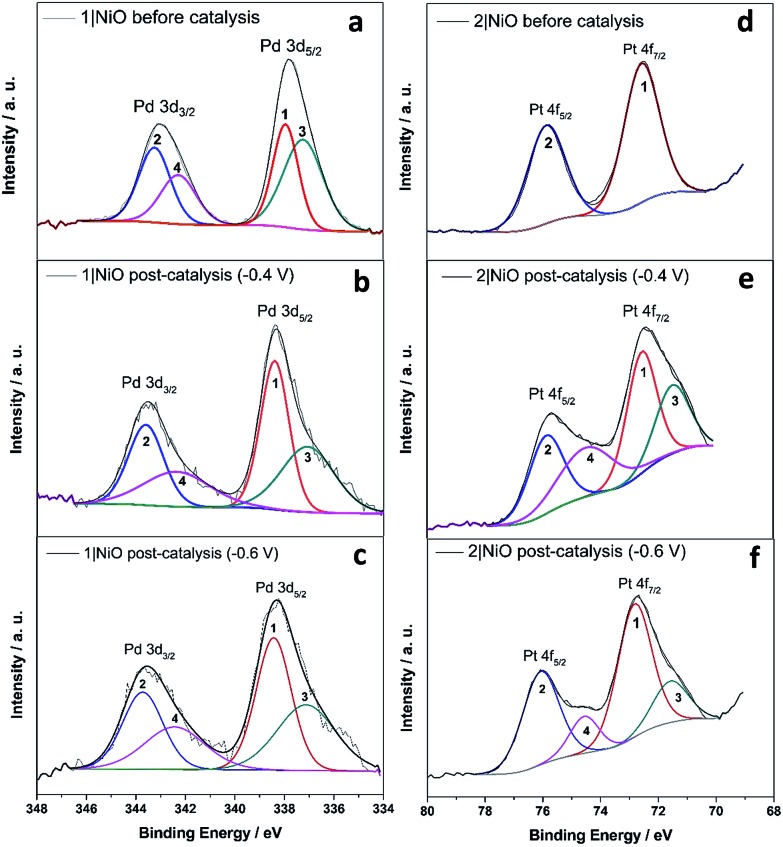
Pd 3d XPS spectrum of **1**|NiO (a) before photoelectrocatalysis, after photoelectrocatalysis under (b) *E*_appl_ = –0.4 V and (c) *E*_appl_ = –0.6 V; Pt 4f XPS spectrum of **2**|NiO (d) before photoelectrocatalysis, after photoelectrocatalysis under (e) *E*_appl_ = –0.4 V and (f) *E*_appl_ = –0.6 V *vs.* Ag/AgCl.

### Electrode characterisation pre- and post-catalysis

The Time-of-Flight Secondary Ion Mass Spectrometry (ToF-SIMS) analysis was carried out on **1**|NiO and **2**|NiO pre- and post-catalysis (Fig. S11–S18 in the ESI†). Higher mass molecular ions corresponding to **1** and **2** were present in the SIMS spectra of as-deposited **1**|NiO and **2**|NiO (pre-catalysis), respectively. This is consistent with the successful adsorption of photocatalysts **1** and **2** on the surface of the nanostructured NiO electrode. Post-catalysis, there was some evidence for the presence of phthalate buffer residues with the detection of a fragment ion at *m*/*z* = 121 due to the PhCO_2_^–^ species. We did not observe any desorption of the photocatalyst in the presence of the buffer (see above) but the results suggest that non-competitive co-adsorption of some phthalate is possible.

The SIMS spectra, 1020–1140 mass per unit, of **1**|NiO before and after photoelectrocatalysis are presented in Fig. 9 and contain the higher mass molecular ions corresponding to dye-catalyst assembly **1**. This indicates that **1** did not decompose during the experiment and is intact on the surface of the NiO during the photoelectrocatalysis under *E*_appl_ = –0.4 V *vs.* Ag/AgCl. The SIMS spectra for dye-catalyst assembly **2**, also contain peaks corresponding to higher mass molecular ions in the post-catalysis samples (Fig. S18 in the ESI†). The intensity of these peaks diminishes going from *E*_appl_ = –0.2 V to –0.6 V *vs.* Ag/AgCl. This indicates that **2** has partially detached or decomposed during photoelectrocatalysis. In Fig. 9, several peaks are present in the range corresponding to dye-catalyst assembly **1**. Inspection of the isotope patterns reveals that there is a combination of H_2_O, Cl^–^ and CH_3_CN coordinated to the Pd catalytic centre. Possible identities of these species are provided in the figure caption.

X-ray Photoelectron Spectroscopy (XPS) measurements were carried out on **1**|NiO and **2**|NiO pre- and post-catalysis (*E*_appl_ = –0.4 V and *E*_appl_ = –0.6 V *vs.* Ag/AgCl). The results are compared in Fig. S5–S10 in the ESI.† There were no significant differences between the Ni 2p XPS spectra of pre- and post-catalysis samples (Fig. S8 and S9 in the ESI†), which indicates that there are no substantial changes to the NiO electrode during the experiments. In particular, the Ni^3+^ to Ni^2+^ ratio was similar and there was no visible Ni^0^ peak at lower binding energy, which has been reported elsewhere for post- catalysis samples.^34^ The binding energy for Ni metal 2p_3/2_ peak on Ni and NiO samples has been reported at 852.6 eV.^35^,^36^

For **1**|NiO, the C 1s and Ru 3d regions (Fig. S5 and S6†) were largely unchanged, confirming that the dye-catalyst assembly **1** is present on NiO before and after photoelectrocatalysis. In both **1**|NiO and **2**|NiO samples, the binding energies for the Ru 3d spin–orbit doublet peaks are situated at 281.1 eV and 285.3 eV for Ru 3d_5/2_ and Ru 3d_3/2_, respectively, which is consistent with reported values for ruthenium tris(bipyridine) electrocrafted on boron doped diamond electrode (Ru 3d_5/2_: 281.3 eV, Ru 3d_3/2_: 285.5 eV).^37^ There are no additional peaks present in the Ru 3d region to suggest that the Ru has decomposed. This is consistent with the presence of molecular ion fragments corresponding to [Ru(bpy)_2_(L)]^+^ in the corresponding TOF-SIMS (Fig. S13 and S17†). Therefore, we conclude that the photosensitiser part of both molecules is stable during the photoelectrocatalysis, even at potentials more negative than *E*_appl_ = –0.6 V *vs.* Ag/AgCl.

The Pd 3d spin–orbit doublet in the spectra for **1**|NiO, pre- and post-catalysis, (Fig. 10(a–c)) contain two major components in each band (1, 2, 3 and 4). Component 1 at 338.4 eV under Pd 3d_5/2_ peak is consistent with spectra reported previously for Pd(ii) (PdCl_2_ (ref. 70) and bis(1,2-ethanediamine-*N*,*N*′)dichloropalladium^71^). The 3d_5/2_ binding energy for Pd(0) should lie between 334 eV and 336 eV, but component (3) has a maximum at 337.1 eV.^72^ Therefore, we do not attribute components (3) and (4) to Pd(0). Instead, (3) and (4) could correspond to a Cl-free complex, *e.g.* [Ru(decb)_2_(bpt)Pd(CH_3_CN)]^3+^ (L1d) identified in the ToF-SIMs spectrum in Fig. 9(a).

In contrast, each doublet peak in the Pt 4f spin–orbit doublet in the **2**|NiO, pre-catalysis, spectrum (Fig. 10(d)) is composed of one component, with binding energies for (1) Pt 4f_7/2_ = 72.6 eV and (2) Pt 4f_5/2_ = 75.9 eV. These binding energies are consistent with those reported previously for Pt(ii) iodo-complexes.^35^,^38^ Additional components (3 and 4) are present at lower binding energies in the spectrum for **2**|NiO, post-catalysis, (Fig. 10(e) and (f)). The additional component (3) under Pt 4f_7/2_ doublet peak has the binding energy of 71.6 eV, which is consistent with Pt(0).^36^,^39^,^73^ This indicates that photocatalyst **2** decomposes during the photoelectrocatalysis and metallic Pt is formed on the NiO surface. These findings are consistent with the ToF-SIMS data, which showed the loss of higher mass species on post-catalysis samples of **2**|NiO at *E*_appl_ = –0.6 V *vs.* Ag/AgCl (Fig. S18 in the ESI†). From these results, we reason that during the photoelectrochemical experiments, 2 decomposes to give Pt^0^ on the surface of the electrode and this could drive H_2_ evolution in this system.

## Discussion

The role of the photocathode in a DSPEC is to use the electrons generated by water oxidation at a photocathode to drive reduction of H^+^ to H_2_. The dye-sensitised photoelectrochemical devices reported previously used either co-adsorbed dyes and catalysts^12^ or covalently linked (but electronically de-coupled) metal ion coordination supramolecular dye-catalyst assemblies.^16^,^34^ High photocurrents have been reported elsewhere for an organic dye with an H_2_-evolving catalyst in solution (however, pH 0 was used, which could be problematic when coupled with an oxygen-evolving photoanode).^40^ Our approach, instead, uses integrated photocatalysts.

In solution, the photophysics of the two photocatalysts is distinct, as summarised in Fig. S40.† In photocatalyst **2**, the electron transfer is in the direction of the peripheral ligands, whereas in photocatalyst **1**, the electron transfer is in the direction of the bridging ligand. In the presence of a sacrificial electron donor, *e.g.* triethylamine, **2** performs surprisingly well considering its apparently unfavourable structure and this has been attributed to its long-lived excited state (≈μs).^28^ In contrast, while the structure of **1** favours electron transfer in the direction of the catalytic centre, the excited state decay (≈ns) could compete with diffusion controlled processes such as reductive quenching by triethylamine, and may explain why no hydrogen was observed in the solution studies.

**Fig. 11:**
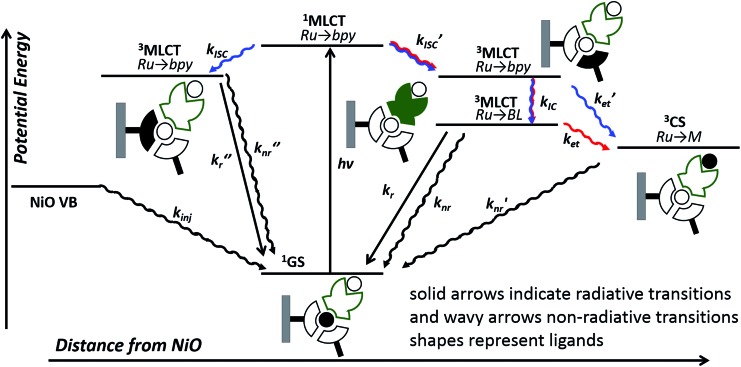
Possible excited state configurations of **1** (red) and **2** (blue) when immobilised on NiO based on the TA and TRIR experiments, DFT and TD-DFT calculations in this work and models proposed in ref. 21, 24, 25 and 28. BL = bridging ligand, bpy = bipyridyl, M = Pt or Pd, CS = charge-separated state.

In this work, we have probed the first steps in photocatalysis using TRIR and TA and demonstrated spectroscopically that rapid photoinduced electron transfer from NiO to the adsorbed photocatalyst occurs. The spectra were complex due to overlapping signals for the charge-separated state and residual excited state in both **1**|NiO and **2**|NiO sets of spectra and multi exponential decay kinetics. This is consistent with studies on model dyes such as, [Ru(4,4′-(CO_2_H)-bpy)_3_]^2+^, [Ru(4,4′-(CO_2_H)-bpy)(bpy)_2_]^2+^ and [Ru(4,4′-(PO_4_H_2_)-bpy)(bpy)_2_]^2+^, reported previously.^24^,^29^,^41^ To help make sense of the possible pathways through which the excited state could evolve, the possible configurations are shown in Fig. 11, which, to simplify the analysis, assumes only one decb ligand is bound. Absorption of visible light, leads to the population of the ^1^MLCT, which rapidly relaxes to the ^3^MLCT, either on the decb ligands (in **2**) or the bridge (in **1**). According to Bräutigam *et al.* a competition between the population of bipyridyl ligands pointing away from the surface (injection-favouring) compared to those anchored to the surface (recombination-favouring) takes place, leading to the observation of a transient absorption spectrum corresponding to the charge-separated state or excited state, respectively.^29^ The TRIR experiments are consistent with this hypothesis, highlighting the loss of symmetry when the dyes are adsorbed onto NiO. In **1**|NiO, electron transfer to the bridge is more favourable than to the peripheral decb ligand, so one would expect that injection would be more favourable in this system. Inspection of the amplitudes for the DAS for the immobilised photocatalysts, in Fig. S30 and S31,† reveals that in **1**|NiO, about 30% of the bleach corresponds to 1*, for **2**|NiO, roughly 50% of the bleach corresponds to 2*. The rapid (<ps) rate of electron transfer from NiO to the photocatalysts, combined with the relative yield compared to residual excited state, implies that this injection process may take place from the ^1^MLCT and the ligand on which this is localised, may determine whether or not charge injection occurs. The absence of a signal corresponding to the reduced photocatalysts at longer timescales (>100–200 ps), despite the long-lived excited species 1*|NiO (*τ* ≈ 1 ns) and 2*|NiO (*τ* ≈ 4 ns), implies that injection does not occur from these ^3^MLCT states.

However, this may not be the whole story under operation of the device. The lifetime of the charge-separated state was found to be extremely short, which is consistent with previous studies of organic dyes on NiO and suggests that recombination between the holes in NiO and the reduced dye dominates.^42^ This implies that photoelectrocatalysis is unfavourable. However, Dillon *et al.* demonstrated that changing the bias applied to the photocathode from +0.4 to –0.4 V *vs.* Ag/AgCl extends the lifetime of a reduced dye, [Ru(4,4′-(PO_4_H_2_)-bpy)(bpy)_2_]^+^, from ps to μs due to filling intra-bandgap states (see inset Fig. 11 for our interpretation). This could both increase the rate of injection and slow down recombination. We have not applied a bias to the films in the TRIR or TA studies reported for **1**|NiO and **2**|NiO, (D'Amario *et al.* estimated a Fermi Level of +250 mV *vs.* Ag/AgCl for NiO prepared under similar conditions), however, a reduction of the rate of recombination is consistent with the increase in photocurrent as *E*_appl_ was increased. The photocurrent onset in our experiments is *ca.* 0.43 V more negative than the valence band edge of NiO. In the TA experiments, in non-aqueous electrolyte under applied bias, by Dillon *et al.*, *ca.* 70% filling of the surface states was estimated at this relative applied potential, giving <60% yield of reduced dye, compared to zero yield when no bias was applied. At *E*_appl_ > –500 mV (where all intra band gap states should be filled), Dillon *et al.* observed a change in kinetics, which they attributed to dye desorption or a build-up of cations at the electrode surface which could stabilise the reduced dye.^24^ This is again consistent with the change in photoelectrochemical behaviour observed in this work for **1**|NiO and **2**|NiO at extreme negative bias (–0.6 V *vs.* Ag/AgCl).

The photocurrent densities for **1**|NiO and **2**|NiO are comparable with the best co-immobilised systems on NiO and ITO. However, **1**|NiO is superior to previously reported photocathodes in terms of the faradaic efficiency and stability of the photocurrent.^10^,^43^ We attribute the high faradaic efficiency of our photocathodes to the direct coupling of the photosensitiser with the catalyst. In the NiO systems reported previously, the dye and the catalyst have been either separate molecules (either co-deposited on the surface or a catalyst in solution) or tethered through non-conjugated linkers.^2^ In such systems, the faradaic yields reported are typically less than 70% and in some cases below 10%, possibly due to slow or inefficient charge-transfer to the catalyst (leading to charging and discharging of the electrode), the presence of side reactions (*e.g.* reduction of O_2_) or the catalyst acting as a redox shuttle by diffusing from the cathode to the anode. Integrating the sensitiser and catalyst within one molecule permits rapid (*i.e.* not diffusion-limited) electron transfer to the catalytic centre. While we do not yet know the precise mechanism for hydrogen evolution in the integrated photocatalyst system, the transient spectroscopy confirms that electron transfer from NiO to the catalyst occurs rapidly upon excitation and the high faradaic yield and stable photocurrents are consistent with catalysis out-competing charge-recombination. Nonetheless, the system is far from optimum at this stage, for example, the light harvesting efficiency of both **1**|NiO and **2**|NiO is low. Thicker and more porous films may improve the performance,^16^ and, in future, chromophores with higher absorption coefficients will be designed to increase the photocurrent density.

We were encouraged by the stability of the Ru chromophore on the NiO surface as, rather than esters, carboxylic acid or phosphonic acid anchoring groups are generally used to adsorb the dye, which present challenges in synthesis and purification^5^,^10^,^12^,^13^,^16^,^17^ Slight changes in the Pd and Pt catalyst structure during operation were anticipated, *e.g.* replacement of the halide ligands with solvent. Kaeffer *et al.*, for example, reported halide ligand substitution (Br^–^ for Cl^–^) in cobalt-diimine–dioxime complexes post-catalysis in their co-immobilised system.^26^ These results are consistent with reactivity at the catalytic metal centre. The reduction of the Pd(ii) centre is expected to be accompanied by dissociation of a chloride anion.^44^,^45^

Initial inspection of the photocurrent density and volume of H_2_ produced, suggested that **2**, containing the Pt catalyst, outperformed **1**, which contains the Pd catalyst. However, the increase in current observed (particularly at the most negative applied bias) during irradiation indicated that the electrode surface was changing during the experiment. The XPS and ToF-SIMS results confirm that the Pt complex is unstable in water, especially under irradiation at *E*_appl_ = –0.6 V *vs.* Ag/AgCl, and we attribute the increasing current over time to the formation of electrocatalytically active Pt(0) on the NiO surface. It is also possible that the strong transient reductive photocurrents observed for NiO/Ru–Pt (Fig. 7) correspond to the *in situ* reduction of the Pt(ii) complex, generating Pt(0) particles. Du *et al.* have previously reported the formation of colloidal platinum during photocatalytic H_2_ evolution using a system containing platinum(ii) bi- and terpyridyl chloro complexes, in the presence of a sacrificial electron donor (MeOH or triethanolamine) and an electron relay (TiO_2_).^46^ In contrast, the surface analysis experiments showed that **1**, which contained the Pd centre, was present on the NiO surface after 1 h PEC at potentials from *E*_appl_ = –0.2 to –0.6 V *vs.* Ag/AgCl. These results highlight the importance of characterising the electrodes after the catalysis. Whilst *in situ* characterisation of heterogenous catalysts is challenging, hybrid systems containing molecular catalysts furnished with spectroscopic handles present an opportunity to extract mechanistic information.

While the performance of the photocathodes is encouraging, we anticipate that much higher yields are possible with some minor alterations to our experimental setup. Better device engineering is necessary to improve mass transport. The geometry of the custom-built PEC cell (Fig. S1†) is not optimised and bubble formation on the photocathode surface led to a drop in the active area. In this study, no membrane was used to separate the anode and photocathode so the presence of oxygen formed at the Pt anode could provide a recombination pathway. While reasonable photocurrents were observed in pH 7 buffer, pH 3 was optimum for H_2_ evolution. Raising this will be desirable for tandem devices.

## Conclusions

Light driven H_2_ production from water by two new photocathodes, comprised of integrated photocatalysts **1** and **2** adsorbed on NiO, has been described. Stable photocurrents and sustained H_2_ production was observed for both photocathodes immersed in pH 3 buffer by PEC experiments with in-line gas chromatography, in the absence of sacrificial agents. The faradaic efficiencies were estimated between 30–90%, depending on *E*_appl_. Surface analysis experiments revealed that the Ru photosensitiser and the Pd catalyst are stable during photoelectrochemical H_2_ evolution over a range of *E*_appl_, whereas the Pt catalyst decomposed at more negative potentials. These findings were consistent with the observed photocurrent during PEC H_2_ production, which was most stable for **1**|NiO. These results are promising for the development of efficient photoelectrocatalytic devices for storing the energy from sunlight in chemical bonds. Future efforts will be directed towards increasing the absorptivity of the photocathodes and raising the optimum pH through modifications to the ligand structure.

## Experimental

### Materials

3,5-Bis (pyridin-2-yl)-1,2,4-triazole (Hbpt),^47^ 4,4′-di(ethylcarboxy)-2,2′-bipyridine (decb),^48^*cis*-[Ru(decb)_2_Cl_2_],^49^*cis*-[Pt(DMSO)_2_I_2_],^27^*cis*-[Pd(DMSO)_2_Cl_2_], [Ru(decb)_2_(2,5-bpp)](PF_6_)·2H_2_O, and [Ru(decb)_2_(2,5-bpp)PtI(CH_3_CN)](PF_6_)_2_,^27^ were prepared using literature methods, solvents obtained were used without further purification.

### [Ru(decb)_2_(Hbpt)](PF_6_)_2_·2H_2_O

[Ru(decb)_2_(Hbpt)](PF_6_)_2_·2H_2_O was prepared using a modified literature method.^50^ 84 mg (0.37 mmol) of 3,5-bis(2-pyridyl)-4-hydro-1,2,4-triazole(Hbpt) were dissolved in 60 ml EtOH/water (3 : 1) and heated for 20 minutes. 200 mg (0.26 mmol) of [Ru(decb)_2_Cl_2_] in ethanol was added slowly over 30 minutes. The reaction was refluxed for a further 6 h, with a total volume of 100 ml. The ethanol was removed *in vacuo*. Following this, 40 ml of water was added to the reaction mixture. The red aqueous reaction mixture was filtered and an aqueous solution of NH_4_PF_6_ was added in excess to the filtrate and a precipitate formed. The precipitate was washed with diethyl ether and collected by filtration. For further purification, the filtrate was recrystallized with acetone/H_2_O (3 : 1), yielding a black/brown solid. Yield: 212 mg (0.17 mmol, 65%). ^1^H-NMR (400 MHz, DMSO-d6) *δ*[ppm] = 1.30–1.40 (m, 12H), 4.39–4.49 (m, 8H), 7.39 (dd, 1H), 7.49–7.67 (m, 2H), 7.84 (dd, 1H), 7.93–8.25 (m, 8H), 8.33 (d, 1H), 8.62 (d, 1H), 9.17–9.40 (m, 4H). Elemental analysis for C_44_H_45_F_12_N_9_O_10_P_2_Ru calc.: C 42.2%, H 3.6%, N 10.1%. Found: C 42.12%, H 3.18% and N 10.28%.

### [Ru(decb)_2_(bpt)PdCl(H_2_O)](PF_6_)_2_·2H_2_O

28 mg (0.11 mmol) of *cis*-Pd(DMSO)_2_Cl_2_ and 65 mg (0.05 mmol) of [Ru(decb)_2_(Hbpt)](PF_6_)_2_·2H_2_O were added to 15 ml of hot EtOH and stirred under nitrogen until dissolved. The reaction was brought to reflux temperature for 24 h. A precipitate formed and the solution was allowed to cool to room temperature. The precipitate was collected by vacuum filtration and washed with cold EtOH and diethyl ether. Yield: 58 mg (0.04 mmol, 84%). ^1^H-NMR (400 MHz, acetonitrile-d3) *δ*[ppm] = 1.35–1.45 (m, 12H), 4.39–4.49 (m, 8H), 7.29 (t, 1H), 7.50 (d, 2H), 7.65 (t, 1H), 7.70 (d, 1H), 7.75–7.82 (m, 3H), 7.83 (d, 1H), 7.89 (d, 1H), 7.95–8.05 (m, 2H), 8.27 (d, 1H), 8.36 (t, 1H), 8.51 (d, 1H), 8.95–9.08 (m, 3H), 9.18 (d, 1H), 9.60 (d, 1H), 11.12 (d, 1H). Elemental analysis for C_44_H_46_F_12_N_9_O_11_P_2_RuPdCl calc.: C 37.48%, H 3.2%, N 8.94%. Found: C 37.08%, H 2.71% and N 9.03%.

### Photocathode preparation

Mesoporous NiO photocathodes were prepared by following a reported procedure.^51^ A NiCl_2_ precursor solution was prepared by dissolving anhydrous NiCl_2_ (1 g) and the tri-block co-polymer F108 (poly (ethylene glycol)-*block*-poly (propylene glycol)-*block*-poly (ethylene glycol)) (1 g) in ethanol (6 g) and ultrapure Milli-Q water (3 g). The precursor solution described above was spread onto fluorine doped tin oxide (FTO) conducting glass substrates (Pilkington TEC15, sheet resistance 15 Ω per square) using Scotch tape as a spacer (0.79 cm^2^), followed by sintering in an oven at 450 °C for 30 min. Undyed NiO films were prepared to a thickness of 1.5 μm, measured using a Bruker DektakXT stylus profilometer and averaged over 5 samples. Dye sensitised electrodes **1**|NiO and **2**|NiO were prepared by soaking the NiO electrodes in acetonitrile solutions of the dye-catalyst assemblies **1** and **2** (0.3 mM) for 16 h at room temperature.

### Optical and IR spectroscopy

The ultraviolet-visible (UV-vis) absorption spectra of the dye-catalyst assemblies in solution and adsorbed on nanostructured NiO films were recorded using a Shimadzu 1800 UV-vis spectrophotometer. The infrared spectra were measured on a Varian FTS 800 FT-IR spectrometer. Solid samples of sensitised NiO films were removed from the FTO substrate with a spatula and were mixed with potassium bromide (99%, spectroscopic grade, Fisher Scientific) using a pestle and mortar and pressed into disks.

### Electrochemical and photoelectrochemical experiments

Electrochemical (EC) and photoelectrochemical (PEC) measurements were carried out using an IviumStat potentiostat. Electrochemical measurements were conducted on the dye-sensitised nanostructured NiO photocathodes using a custom-made three-electrode photoelectrochemical cell (Fig. S1 in ESI†). A platinum wire was used as the counter electrode. Ag/AgCl (3.0 M NaCl, *E*oAg/AgCl = 0.210 V *vs.* NHE) was used as the reference electrode for the measurements in aqueous solutions, where of 0.1 M potassium hydrogen phthalate and 0.1 M KCl were used as a supporting electrolyte. All potentials are reported *vs.* Ag/AgCl reference electrode. For conversion to NHE, the following equation was used: *E*_NHE_ = *E*_Ag/AgCl_ + *E*oAg/AgCl. For conversion to RHE, Nernst equation was used (*E*_RHE_ = *E*_Ag/AgCl_ + 0.059 pH + *E*oAg/AgCl). During the PEC experiments, irradiation was provided by a 300 W Xe lamp (Oriel) fitted with an AM1.5 filter (Newport). This was calibrated using a calibrated reference solar cell (Newport) to give a power density of 100 mW cm^–2^ (1 sun) at the photocathode surface with the irradiated area of 0.79 cm^2^. The cell was degassed with Ar for at least 20 minutes prior to each measurement. The pH was measured using a pH mV^–1^ Benchtop Meter (Hanna instruments). During chronoamperometry measurement chopped light illumination was first applied with 30 s intervals (1 cycle: 30 s light on/30 s light off), which was followed by constant light illumination. Before every PEC measurement, the working electrode (WE) was held 30 s to 1 min under the potential applied during the measurement, *E*_appl_, in the dark to stabilise the background current. The faradaic efficiency, *η*_Far_, was calculated by dividing the amount of H_2_ produced experimentally (mol) with the theoretical H_2_ production (mol) according to the charge generated from the photocurrent. The results from a control experiment with Ru(dcbpy)_3_Cl_2_ (dcbpy = 2,2′-bipyridyl-4,4′-dicarboxylic acid) *E*_appl_ = –0.4 V *vs.* Ag/AgCl is provided in Fig. S35.†

### Ultrafast transient absorption spectroscopy

Picosecond Transient Absorption and Time-Resolved Infra-Red (TRIR) spectra were recorded using the ULTRA instrument located in the Central Laser Facility at the Rutherford Appleton Laboratory. Briefly, two Ti: sapphire amplifiers of 10 kHz and 1 kHz were synchronized using a common 65 MHz oscillator. The 1 kHz output was used as a pump and the 10 kHz as probe. The pump laser was tuned to 470 nm by optical parametric amplification (OPA, Light Conversion, TOPAS). For TAS, the probe pulse was provided by a white light continuum (WLC), which was generated by focusing 800 nm into CaF_2_. The mid-IR probe pulses were generated using OPA with difference frequency mixing. The IR probe beam was split to form reference and probe beams which were passed through spectrographs onto MCT array detectors (IR Associates). High speed data acquisition systems (Quantum Detectors) allowed 10 kHz acquisition and processing of the probe and reference pulses to generate a pump-on pump-off infrared absorption difference signal. Spot sizes in the sample region were *ca.* 150 and 50 μm for the pump and probe, respectively, with a pump energy of 50 nJ. For all measurements, the pump polarization was set to magic angle relative to the probe.

Samples were prepared by adsorbing the dye on a mesoporous NiO film deposited on a CaF_2_ window (Crystran). The NiO films were prepared by spraying a saturated solution of NiCl_2_ in acetylacetone onto the surface of the CaF_2_ window, which was preheated to 450 °C on a hot plate; this was then allowed to cool slowly to room temperature to give a compact film of NiO. The mesoporous layer was then deposited on top of the compact layer using the F108-templated precursor solution described above; the excess was removed by doctor blade. The film was sintered at 450 °C for 30 min, and an additional layer of precursor solution was applied and sintered to increase the film thickness. All spectra were recorded in IR cells (Harrick Scientific Products Inc.) with CaF_2_ windows. For samples prepared in solution, a 200 μm path length was used. In all experiments, the cell was rastered in the two dimensions orthogonal to the direction of beam propagation to minimize localized sample decomposition.

### Gas chromatography

Gas chromatography (GC) measurements were carried out using a Shimadzu chromatograph with the thermal conductivity detector (TCD) operating at 50 °C and fitted with a ShinCarbon ST Micropacked column (Restek) using Ar as a carrier gas. The experimental setup for the in-line, continuous gas sampling and analysis method is described in full by Summers *et al.*^52^

Briefly, Ar was continuously flowed through the electrolyte solution and into a 6-port, 2-position switch (VICI) at a constant flow (typically 10 cm^3^ min^–1^). Flow from the PEC cell was directed through a cold trap to a rheodine switch which injected a gas samples to the GC at three-minute intervals, maintained using a mass flow controller (Bronkhorst, E-Flow series). A 200 μl sample was analysed automatically every 3 min. The calibration of the amount of H_2_ detected was carried out by varying the flow rate of H_2_ (mol min^–1^) into the system using the 6-port, 2-position switch. The H_2_ peak area varied linearly with the flow rate and the peak area was plotted against the flow rate on a calibration plot. The gradient of the line was used to calculate the H_2_ production rate (mol min^–1^) and to get the total amount of H_2_ produced during an experiment, the production rate was integrated with respect to time.

### X-ray photoelectron spectroscopy

X-ray photoelectron spectroscopy (XPS) was carried out on Kratos Axis Nova XPS spectrometer using a monochromatic Al Kα source. The samples were mounted on a clean aluminium platen and immobilised using double sided adhesive tape. The largest analysis area available in this spectrometer (300 μm × 700 μm) was used. All the measurements were repeated on three different analysis positions with non-overlapping analysis areas. Charge compensation was used throughout the measurements. Spectra were analysed using CasaXPS software^53^ (version 2.3.16). Gaussian (70%) Lorentzian (30%), defined in CasaXPS as GL(30), profiles were used for each component. Spectra have been calibrated to obtain the adventitious C 1s spectral component binding energy of 284.7 eV.

### Time-of-flight secondary ion mass spectrometry

Static SIMS analyses were carried out using an ION-TOF ‘TOF-SIMS IV – 200’ instrument (ION-TOF GmbH, Münster, Germany) of single-stage reflectron design.^54^ Positive and negative ion spectra and images of the samples were obtained using a Bi_3_^2+^ focused liquid metal ion gun at 25 keV energy, incident at 45° to the surface normal and operated in ‘bunched’ mode for high mass resolution. This mode used 20 ns wide ion pulses at 6.7 kHz repetition rate. Charge compensation was effected by low-energy (*ca.* 20 eV) electrons provided by a flood gun. The total ion dose density was less than 1 × 10^16^ ions per m^2^. The topography of the sample surface and the ion gun mode of operation limited the mass resolution in this work to *ca. m*/Δ*m* = 5000. Positive and negative ion static SIMS spectra and images were recorded from the samples at room temperature. Raw data containing the secondary ions recorded at each pixel was acquired with a 128 × 128 pixel raster and a field of view of 200 μm × 200 μm. The samples for ToF-SIMS analysis were mounted directly onto a sample holder using small pieces of silicone-free double-sided tape (3M grade 665).

### Quantum chemical methods

Quantum chemical methods were used to model the electronic structure of the ground state singlet and lowest energy triplet states of three model systems [Ru(bipy)_2_(bpt)PdCl](PF_6_)_2_ [Ru(dmcb)_2_(bpt)PdCl](PF_6_)_2_, and [Ru(dmcb)_2_(bpt)PdCl(H_2_O)](PF_6_)_2_. Density functional theory (DFT) modelled the ground-state structures (singlet and triplet) and time-dependent density functional (TD-DFT) methods were used to characterise the low-lying electronic excited states. Either the hybrid B3LYP^55^–^57^ or its Coulomb adjusted variant cam-B3LYP^58^ was used with the double-zeta quality LanL2DZ basis set.^59^–^62^ However, the B3LYP functional performed better than the cam-B3LYP in modelling the energies of the low-lying singlet excited states and thus the simulated UV/vis spectra were a closer fit to the experimental. For this reason, the results obtained using the B3LYP are reported here.

The same general approach was used for all complexes. Initial structures were generated by molecular mechanics methods and these were optimised at the B3LYP/LanL2DZ model chemistry. The stability of the wavefunction was then tested followed by calculation of the vibrational frequencies. Absence of imaginary frequencies confirmed that all optimised structures were located at a minimum on their respective potential energy hypersurface. Because of difficulties with optimising structure containing ethylester functional groups, the model systems chosen contained either unsubstituted bipy ligand or the dimethylcarbonylate substituted ligands (dmcb). These allowed the effect that ester functional groups have on the electronic structures of the excited states of these systems to be estimated. Calculations were performed in the presence of either acetonitrile or water as indicated using the PCM (Polarisable Continuum Method) approach.^63^–^65^

All calculations were performed using the Gaussian 16, Revision A.03 (ref. 66) programme suite. Molecular structures and electron-density difference maps were visualised using GaussView 03.^67^ Orbital contributions for excited states and simulations of UV/vis. Spectra were obtained using AOMix version 6.88.^68^,^69^ Calculations were performed on the Fionn system at the Irish Centre for High End Computing.

## Conflicts of interest

There are no conflicts to declare.

## Supplementary Material

Supplementary information
