# Supplementary material for: Photoelectrocatalytic H2 evolution from integrated photocatalysts adsorbed on NiO
Source: Chem Sci. 2018 Oct 4;10(1):99–112. doi: 10.1039/c8sc02575d (PMC6333170; doi:10.1039/c8sc02575d)
Supplement: Supplementary file 1 [file SC-010-C8SC02575D-s001.pdf]

Electronic Supplementary Material (ESI) for Chemical Science.
This journal is © The Royal Society of Chemistry 2018

Electronic Supplementary Information

Photoelectrocatalytic H_2 Evolution from Integrated Photocatalysts Adsorbed on NiO

Nils Põldme[a], Laura O'Reilly[b], Ian Fletcher[c], Jose Portoles[c], Igor V. Sazanovich[d], Michael Towrie[d], Conor Long[b], Johannes G. Vos[b], Mary T. Pryce*[b] and Elizabeth A. Gibson*[a]

[a] School of Natural and Environmental Science, Newcastle University, Newcastle upon Tyne, NE1 7RU, UK.

E-mail: Elizabeth.gibson@ncl.ac.uk

[b] School of Chemical Sciences, Dublin City University, Dublin 9, Ireland.

E-mail: Mary.pryce@dcu.ie

Table of Contents

Table of Contents 1

Photoelectrochemical Characterisation 2

Electrode Surface Characterisation 4

X-Ray Photoelectron Spectroscopy 4

Time-of-Flight Secondary Ion Mass Spectrometry (ToF-SIMS) 8

Quantum Chemical Calculations 14

Transient absorption spectra 19

Absorption spectra pre vs. post-catalysis 23

Control experiment $Ru(dcbpy)_3^{2+}$ 23

Mass spectrometry of **1** 24

FTIR spectra of immobilised photocatalysts 24

References 27

Photoelectrochemical Characterisation

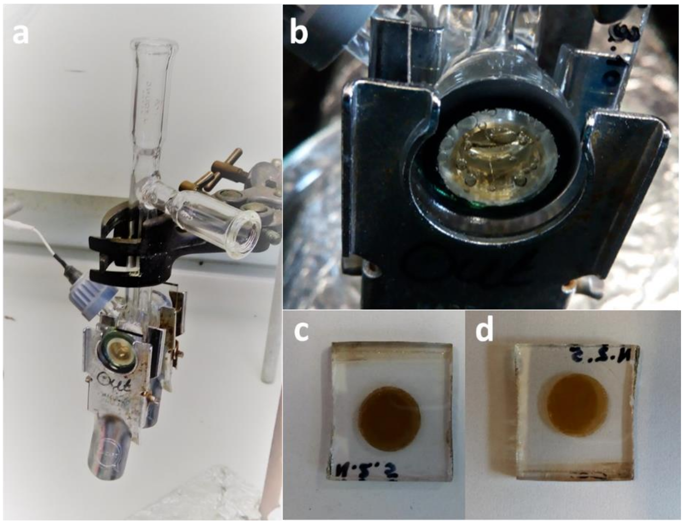

Figure S1. a) Custom built photoelectrochemical cell, b) Hydrogen bubbles forming on **1**|NiO on FTO glass, c) **1**|NiO on FTO glass before photoelectrocatalysis, d) **1**|NiO on FTO glass after photoelectrocatalysis.

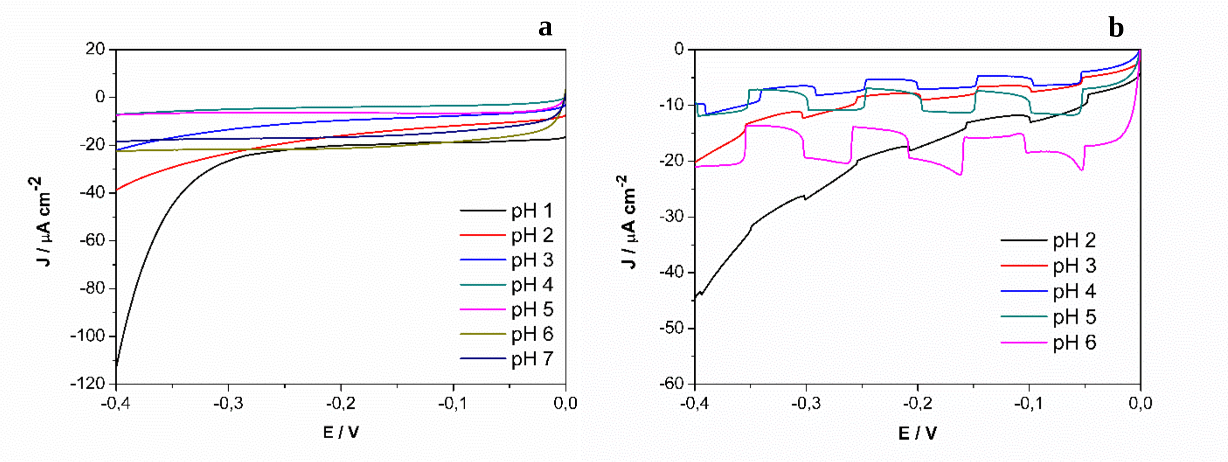

Figure S2. Linear Sweep Voltammetry (LSV) measurements on **1**|NiO in 0.1 M KCl aqueous electrolytes with additions of HCl to adjust the pH from pH1 to pH 7, (a) under constant illumination (V vs. Ag/AgCl) and (b) under chopped light illumination (E / V vs. Ag/AgCl).

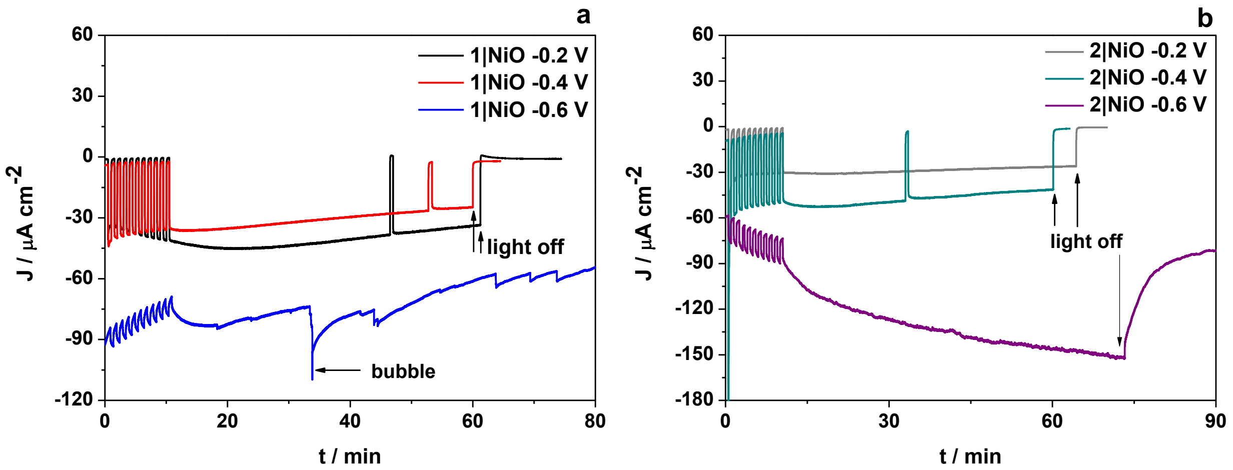

Figure S3. Chronoamperometry measurements of **1**|NiO (a) and **2**|NiO (b) in pH 3 aqueous electrolytes with 0.1 M potassium hydrogen phthalate. E_{appl} = – 0.2 V, – 0.4 V and – 0.6 V vs. Ag/AgCl reference electrode (3.0 M NaCl). Chopped light illumination was applied with 30 s intervals (10 cycles of dark current/photocurrent).

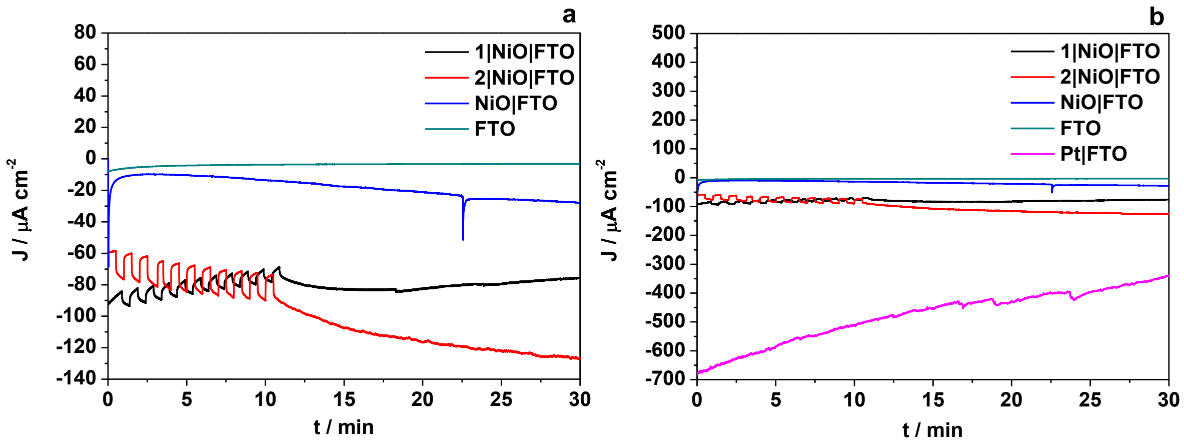

Figure S4. Chronoamperometry measurements of **1**|NiO, **2**|NiO, NiO|FTO, Pt|FTO and bare FTO in pH 3 0.1 M potassium hydrogen phthalate at applied potential of -0.6 V vs. Ag/AgCl reference electrode (3.0 M NaCl). On Figure 5.a. the **1**|NiO, **2**|NiO, NiO|FTO and bare FTO are compared. The same results are also presented on Figure 5.b. along with the Pt|FTP measurement results in same condition and under same applied potential. For **1**|NiO and **2**|NiO chopped light illumination was applied during the first 10 min of the measurement (30 s dark current/30 s photocurrent) followed by constant light illumination.

Electrode Surface Characterisation

X-Ray Photoelectron Spectroscopy

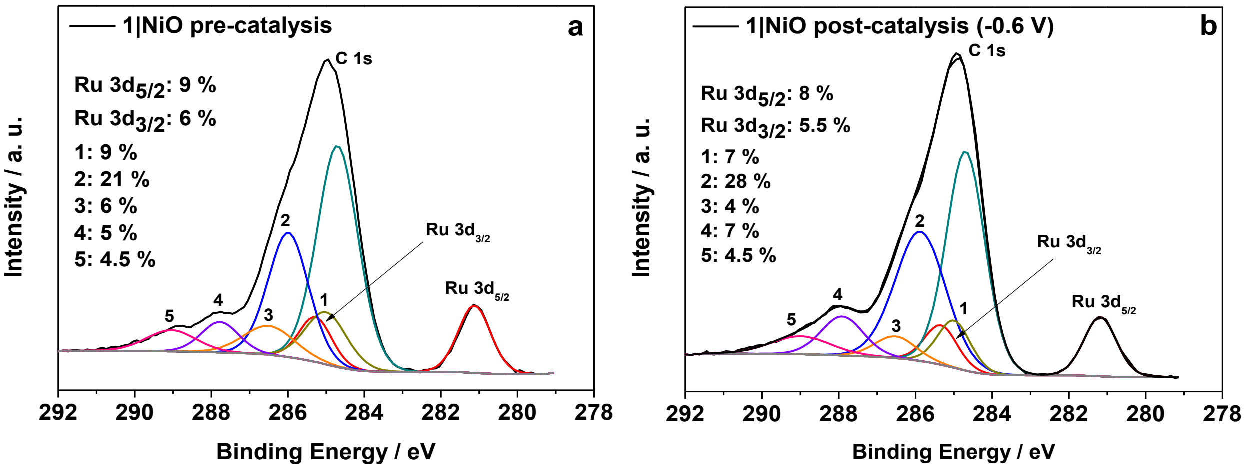

Figure S5. C 1s XPS spectrum of **1**|NiO. (a) - Before photoelectrocatalysis, (b) – after photoelectrocatalysis under -0.6 V (vs. Ag/AgCl ref.) applied potential. The C1s is composed of the following components: (1) – hydrocarbon; (2) – amine; (3) – alcohol, ether; (4) – carbonyl; (5) – ester, acid.[1] Differences between the Ru 3d doublet binding energies of as-deposited and post-catalysis samples are within the error margins (± 0.2 eV).

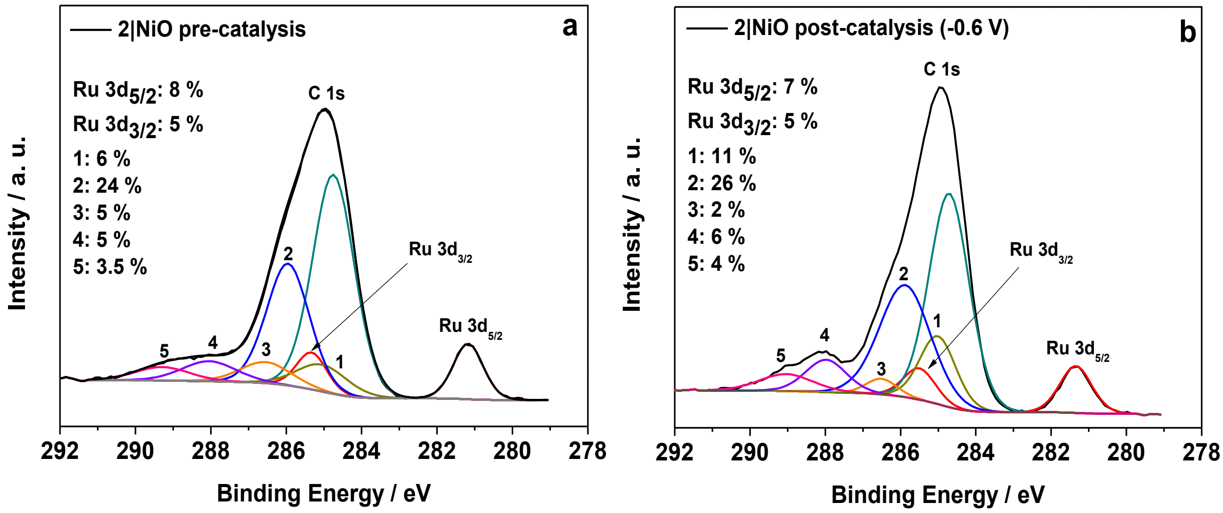

Figure S6. C 1s XPS spectrum of **2**|NiO. (a) - Before photoelectrocatalysis, (b) – after photoelectrocatalysis under -0.6 V (vs. Ag/AgCl ref.) applied potential. (1) – hydrocarbon; (2) – amine; (3) – alcohol, ether; (4) – carbonyl; (5) – ester, acid[1].

Figure S7. O 1s XPS spectrum of **2**|NiO. (a) - Before photoelectrocatalysis, (b) – after photoelectrocatalysis under -0.6 V (vs. Ag/AgCl ref.) applied potential. (1) – oxide, hydroxide; (2) – carbonyl; (3) – ester[1].

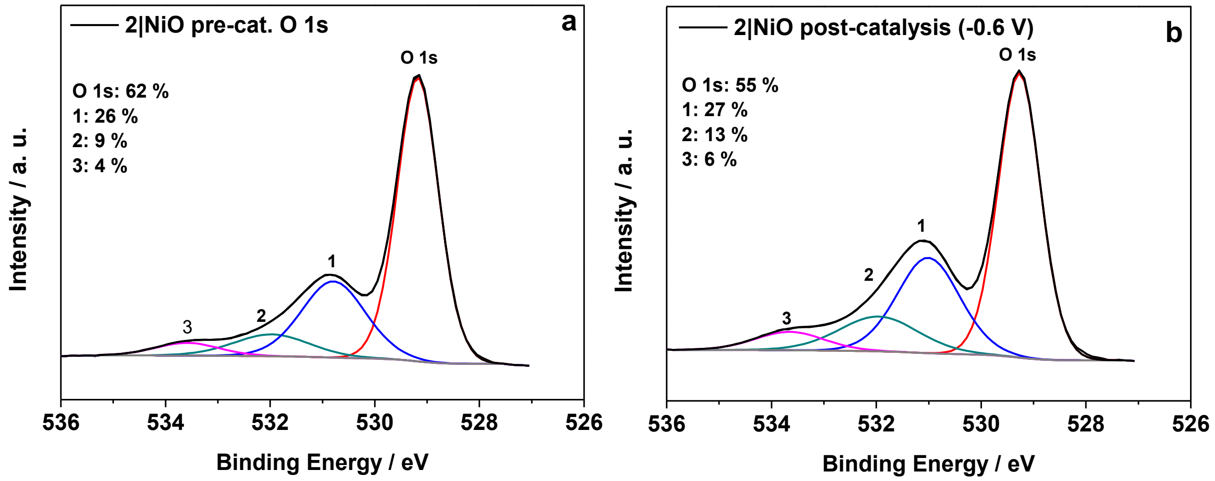

[1]**Figure S8.** Ni 2p XPS spectrum of **1**|NiO. (a) - Before photoelectrocatalysis, (b) – after photoelectrocatalysis under -0.6 V (vs. Ag/AgCl ref.) applied potential. (1) – Ni^{2+}; (2) – Ni^{3+}; (3, 4) – higher energy satellite peaks[2].

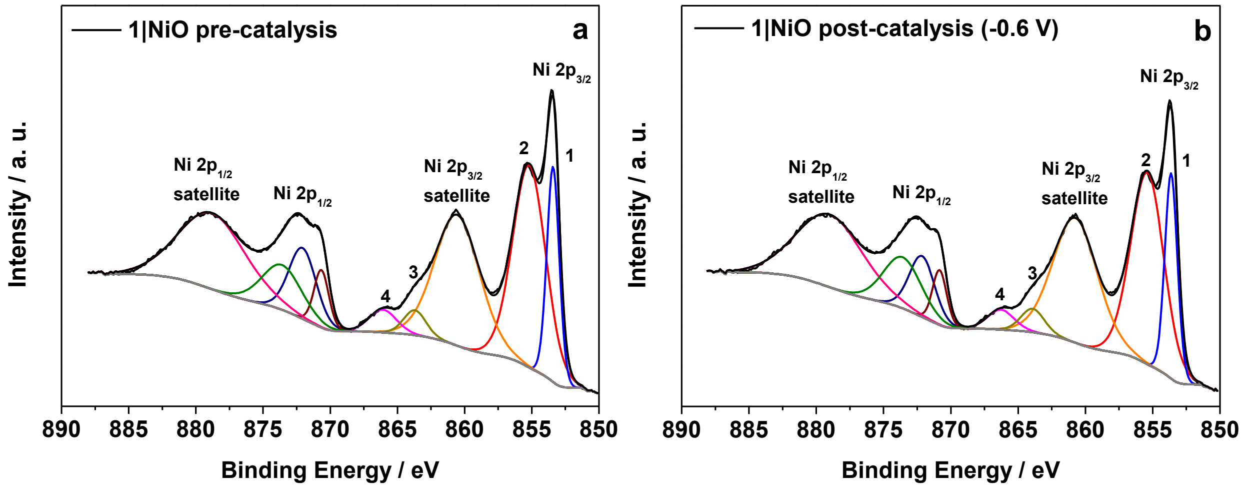

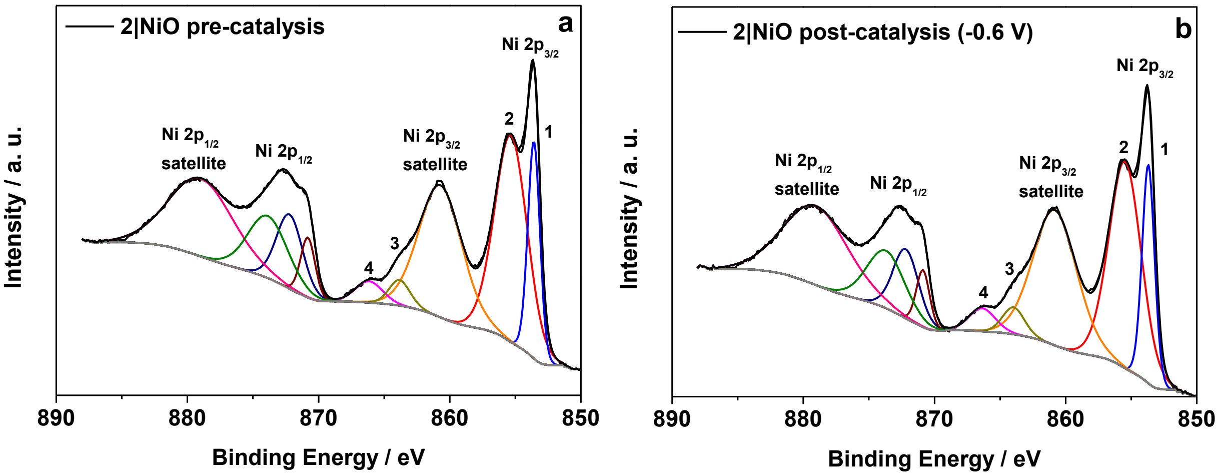

Figure S9. Ni 2p XPS spectrum of **2**|NiO. (a) - Before photoelectrocatalysis, (b) – after photoelectrocatalysis under -0.6 V (vs. Ag/AgCl ref.) applied potential. (1) – Ni2+; (2) – Ni3+; (3, 4) – higher energy satellite peaks[2].

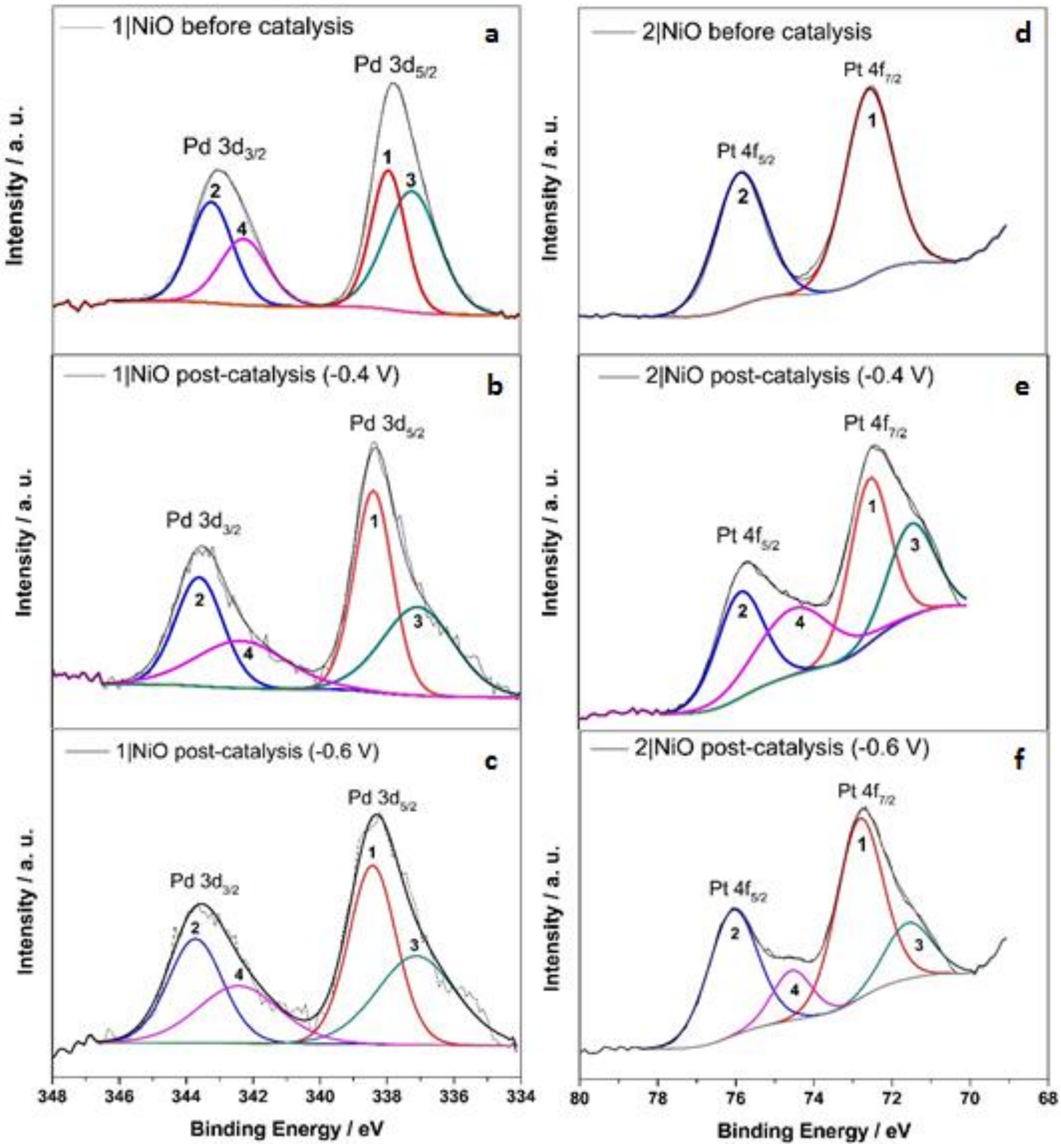

Figure S10. (a) Pd 3d XPS spectrum of **1**|NiO before photoelectrocatalysis, (1, 2) – $PdCl_2$[32],(3, 4) – PdO[33]Pd(I) species[34]. (b) Pd 3d XPS spectrum of **1**|NiO after photoelectrocatalysis under -0.4 V applied potential, (1, 2) – $PdCl_2$[32]; (3, 4) – PdO[33]Pd(I) species[34]. (c) Pd 3d XPS spectrum of **1**|NiO after photoelectrocatalysis under -0.6 V applied potential, (1, 2) – $PdCl_2$[32]; (3, 4) – PdO[33]Pd(I) species[34]. (d) Pt 4f XPS spectrum of **2**|NiO before photoelectrocatalysis, (1, 2) – PtI_2 species[35] (e) Pt 4f XPS spectrum of **2**|NiO after photoelectrocatalysis under -0.4 V applied potential, (1, 2) – PtI_2 species[35]; (3, 4) – Pt[36]. (f) Pt 4f XPS spectrum of **2**|NiO after photoelectrocatalysis under -0.6 V applied potential, (1, 2) – PtI_2 species[35]; (3, 4) – Pt[36].

Time-of-Flight Secondary Ion Mass Spectrometry (ToF-SIMS)

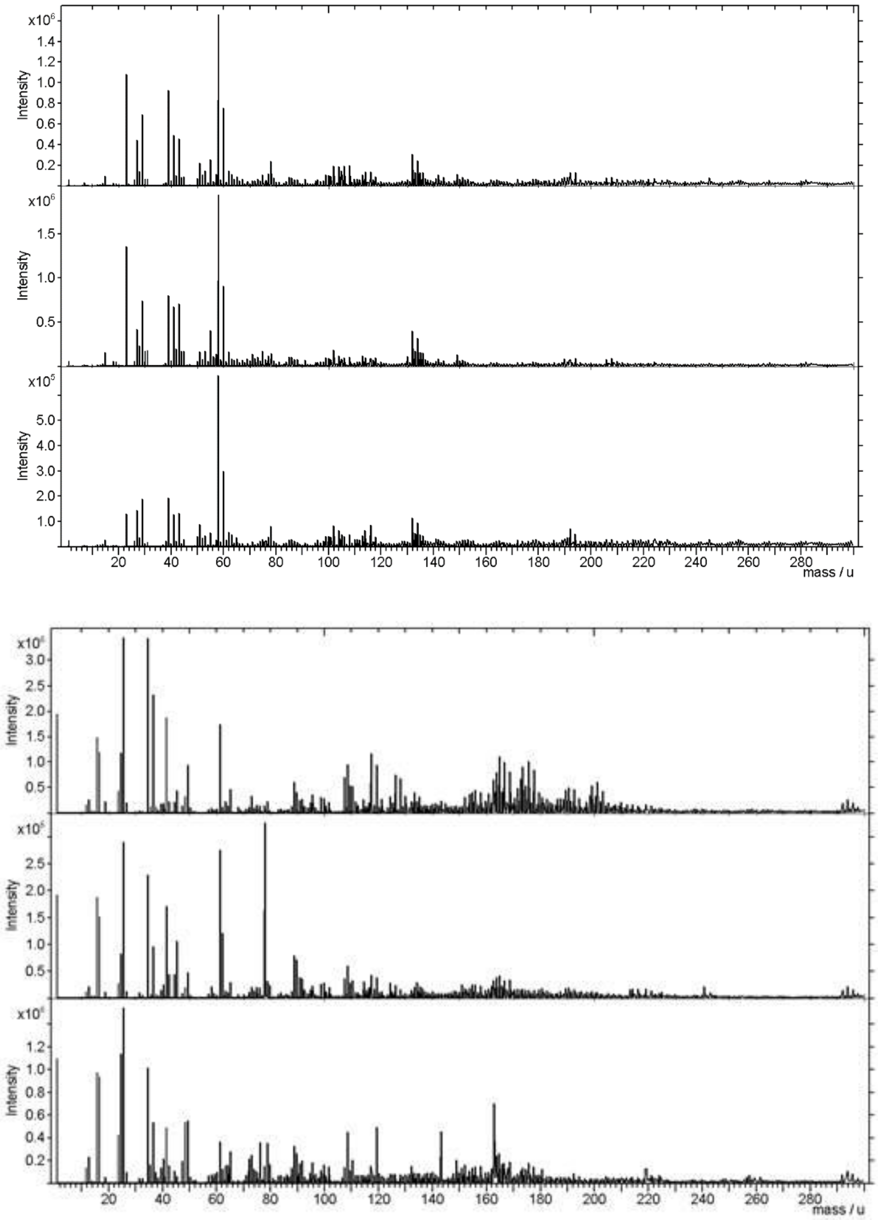

Figure S11. ToF-SIMS positive (A) and negative (B) ion spectra of **1**|NiO taken from three different samples: top:– pre-catalysis, middle: post-catalysis –0.4 V vs. Ag/AgCl, and bottom: post-catalysis at –0.6 V vs. Ag/AgCl. 0 – 300 mass / u range. Ni^+ calcd. m/z = 58.

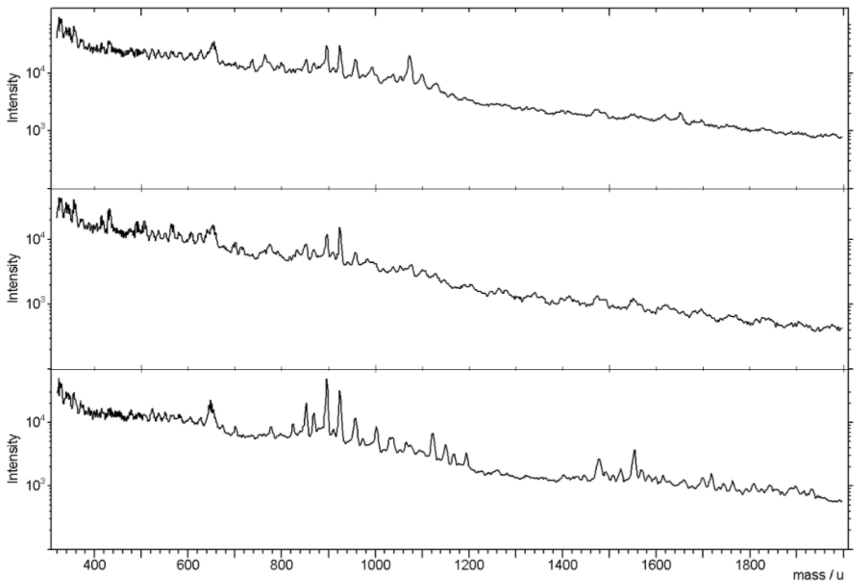

Figure S12. ToF-SIMS positive ion spectra of **1**|NiO taken from three different samples: top:– pre-catalysis, middle: post-catalysis at –0.4 V vs. Ag/AgCl and bottom: post-catalysis –0.6 V vs. Ag/AgCl. 300 – 2000 mass / u range.

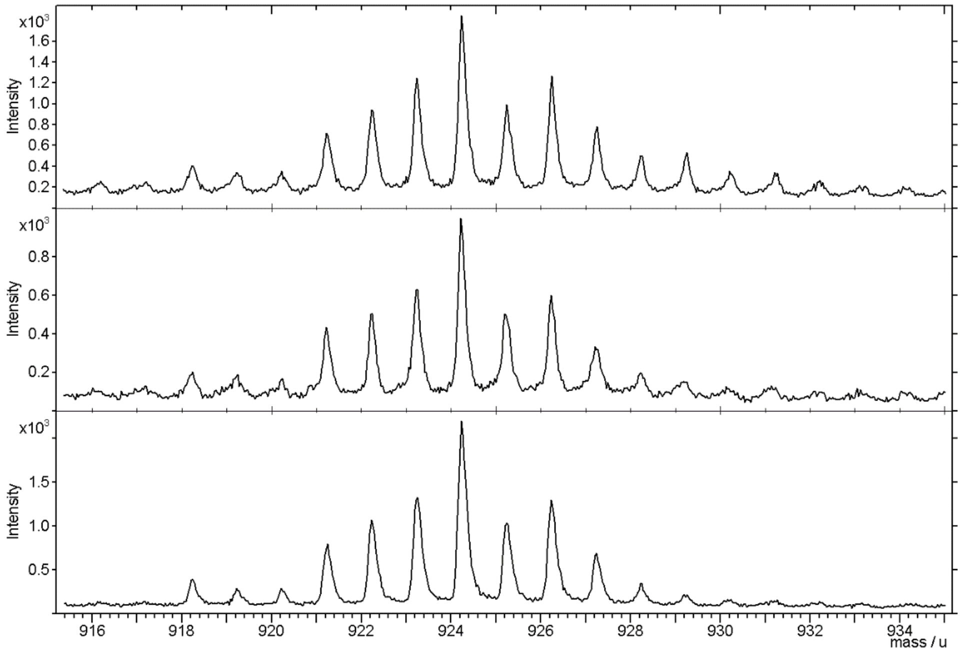

Figure S13. ToF-SIMS positive ion spectra of **1**|NiO taken from three different samples: top:– pre-catalysis, middle: post-catalysis at –0.4 V vs. Ag/AgCl and bottom: post-catalysis –0.6 V vs. Ag/AgCl. 915 – 935 mass / u range. $[Ru(dceb)_2(bpt)]^+$ calcd. *m/z* = 924.

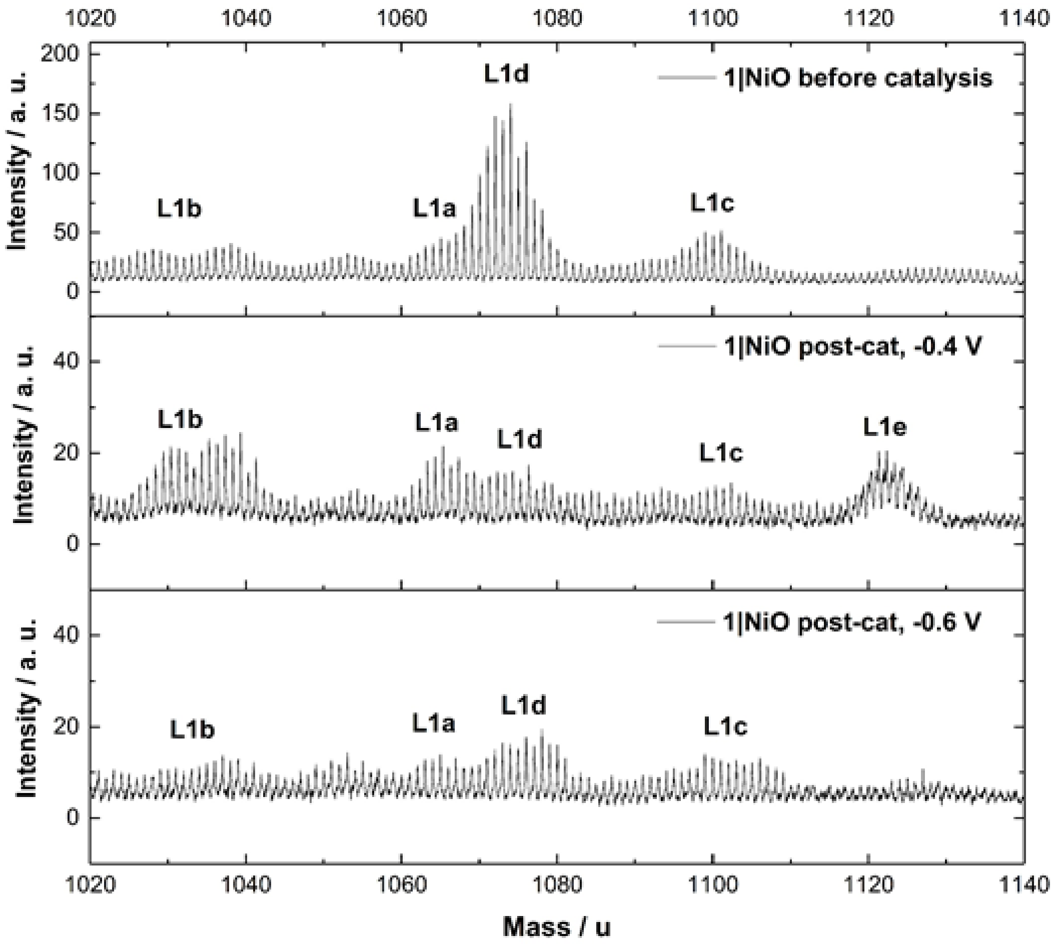

Figure S14. ToF-SIMS positive ion spectra of **1**|NiO taken from three different samples: top: – pre-catalysis, middle: post-catalysis at –0.4 V vs. Ag/AgCl and bottom: post-catalysis –0.6 V vs. Ag/AgCl. 1020 – 1090 mass/u range. L1a - $[Ru(dceb)_2(bpt)PdCl]^{2+}$ calcd. m/z = 1065, L1b - $[Ru(dceb)_2(bpt)Pd]^{2+}$ calcd. m/z = 1030, L1c - $[Ru(dceb)_2(bpt)PdCl(H_2O)_2]^{2+}$ calcd. m/z = 1102, L1d - $[Ru(dceb)_2(bpt)Pd(CH_3CN)]^{2+}$ calcd. m/z = 1071, L1e - $[Ru(dceb)_2(bpt)PdCl(H_2O)_3]^{2+}$ calcd. m/z = 1120.

A

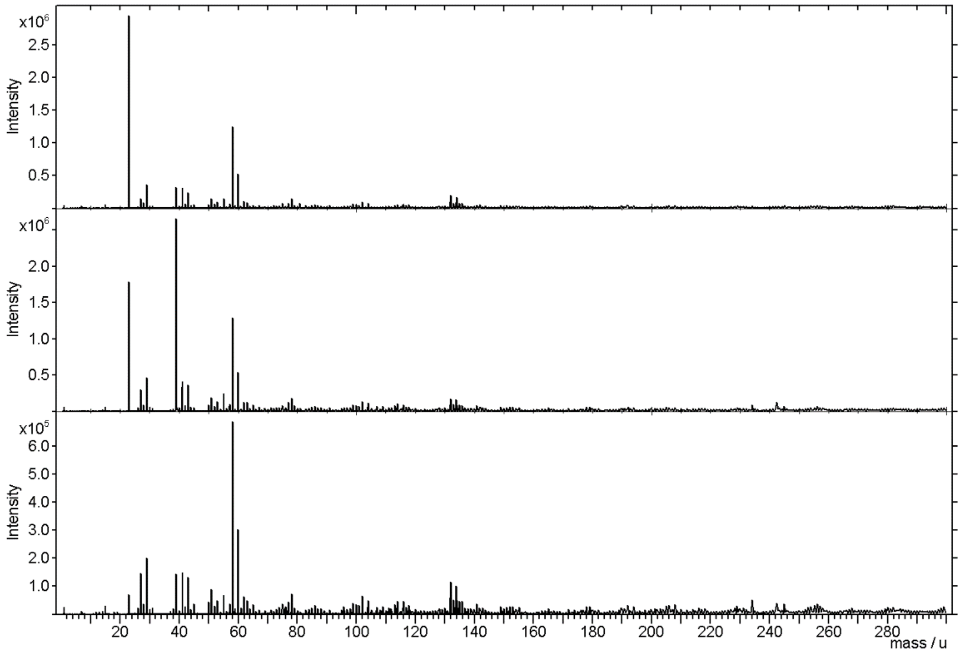

B

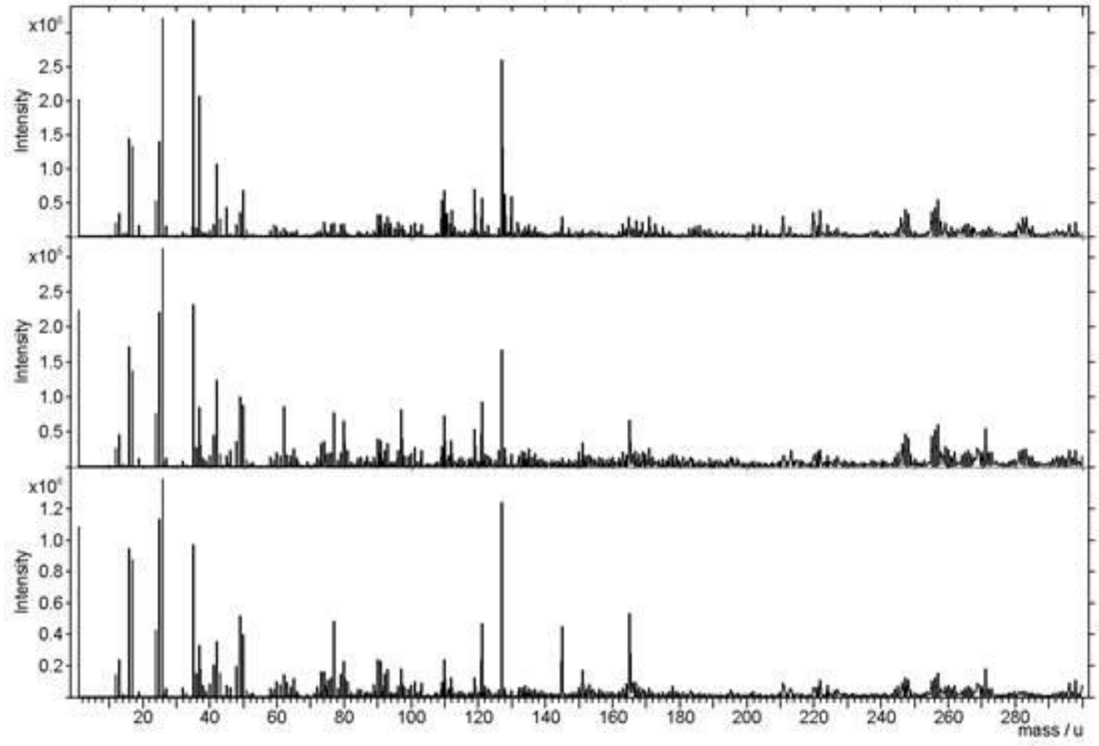

Figure S15. ToF-SIMS positive (A) and negative (B) ion spectra of **2**|NiO taken from three different samples: top:– pre-catalysis, middle: post-catalysis at –0.4 V vs. Ag/AgCl and bottom: post-catalysis –0.6 V vs. Ag/AgCl. 0 – 300 mass / u range. Ni^+ calcd. *m/z* = 58.

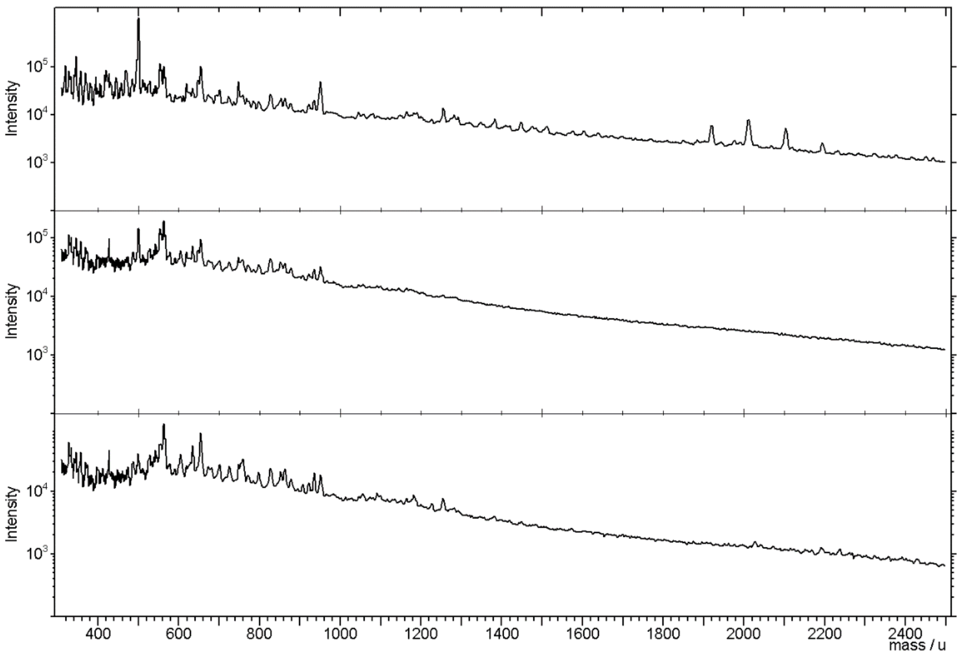

Figure S16. ToF-SIMS positive ion spectra of **2**|NiO taken from three different samples: top:– pre-catalysis, middle: post-catalysis at –0.4 V vs. Ag/AgCl and bottom: post-catalysis –0.6 V vs. Ag/AgCl.300 – 2500 mass / u range.

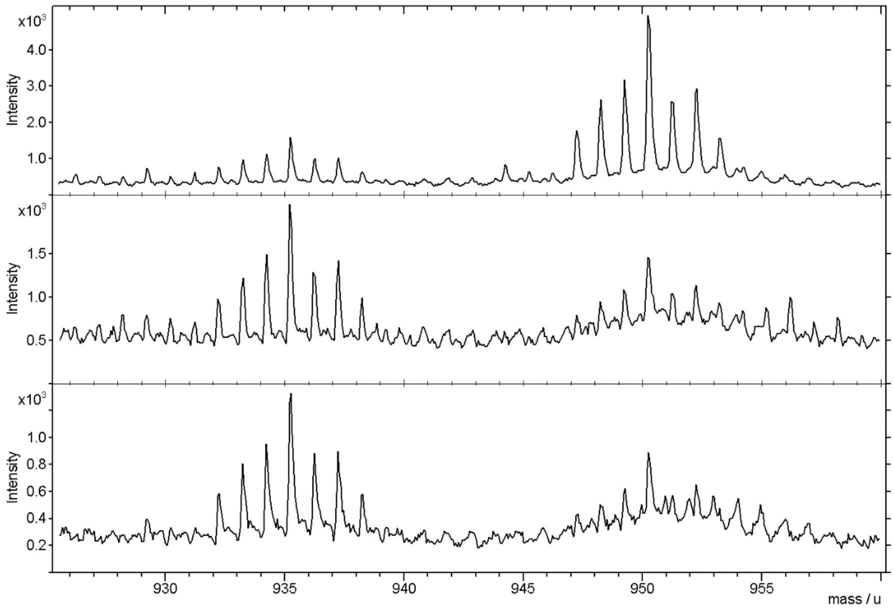

Figure S17. ToF-SIMS positive ion spectra of **2**|NiO taken from three different samples: top:– pre-catalysis, middle: post-catalysis at –0.4 V vs. Ag/AgCl and bottom: post-catalysis –0.6 V vs. Ag/AgCl. 925 – 960 mass / u range. $[Ru(bpy)_2(2,5\text{-bpp})]^{+\bullet}$ calcd. *m/z* = 935, $[Ru(bpy)_2(2,5\text{-bpp})]^{2+}(OH^-)$ = 951.

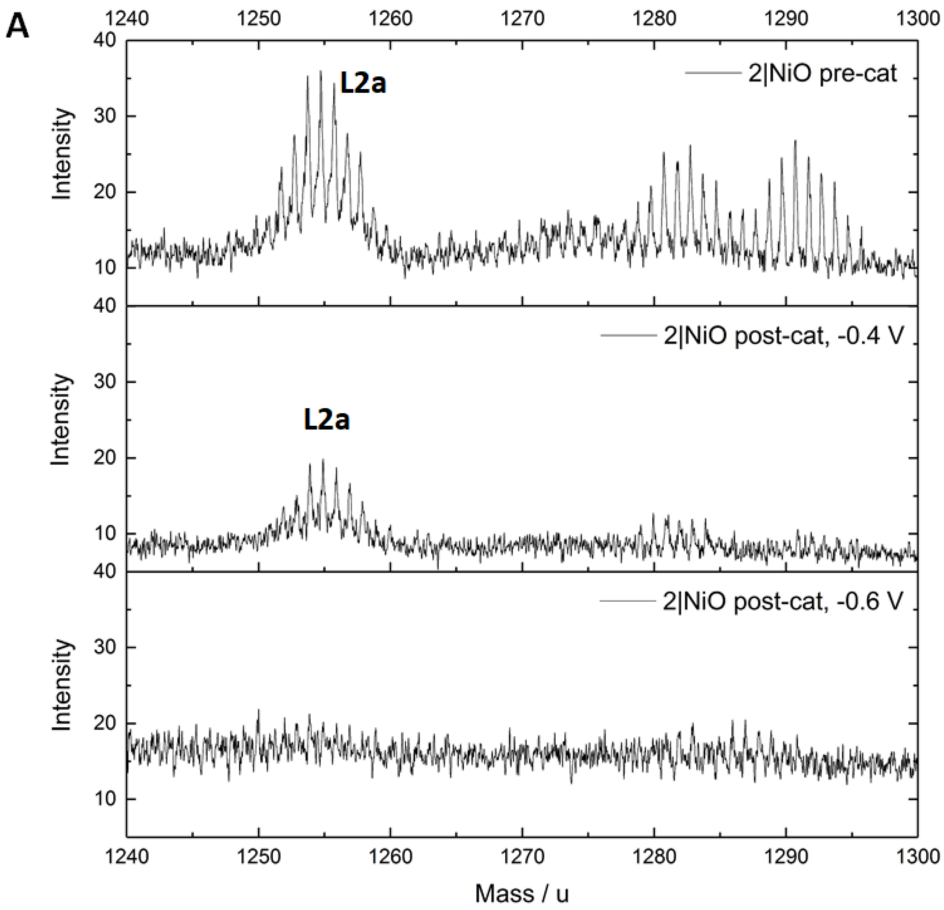

A
L2a
2|NiO pre-cat
L2a
2|NiO post-cat, -0.4 V
2|NiO post-cat, -0.6 V
Intensity
Mass / u

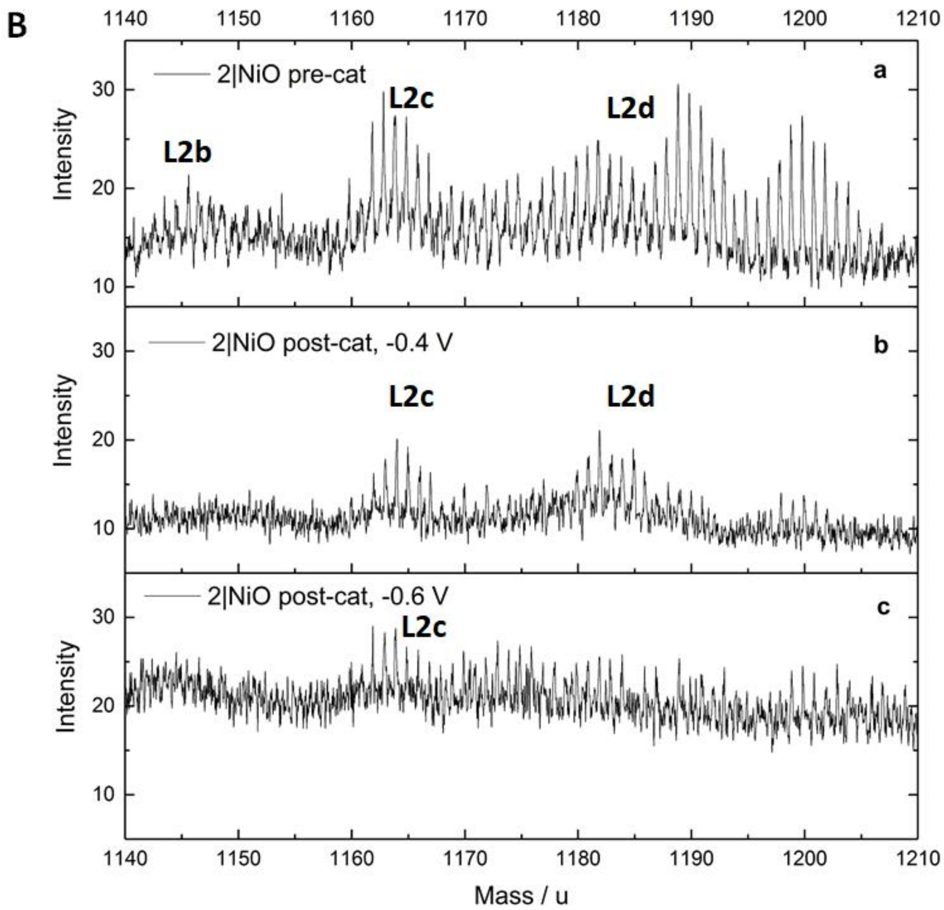

Figure S18. A and **B:** ToF-SIMS positive ion spectra of **2**|NiO taken from three different samples: top:– pre-catalysis, middle: post-catalysis at –0.4 V vs. Ag/AgCl and bottom: post-catalysis –0.6 V vs. Ag/AgCl. 1240 – 1300 mass / u range. **A: L2a -** $[Ru(bpy)_2(2,5\text{-bpp})PtI]^+$ calcd. *m/z* =1255. **B: L2b -** $[Ru(bpy)_2(2,5\text{-bpp})Pt(H_2O)]^+$ calcd. *m/z* = 1146, **L2c -** $[Ru(bpy)_2(2,5\text{-bpp})Pt(H_2O)_2]^+$ calcd. *m/z* = 1164, **L2d -** $[Ru(bpy)_2(2,5\text{-bpp})Pt(H_2O)(CH_3CN)]^+$ calcd. *m/z* = 1187.

Quantum Chemical Calculations

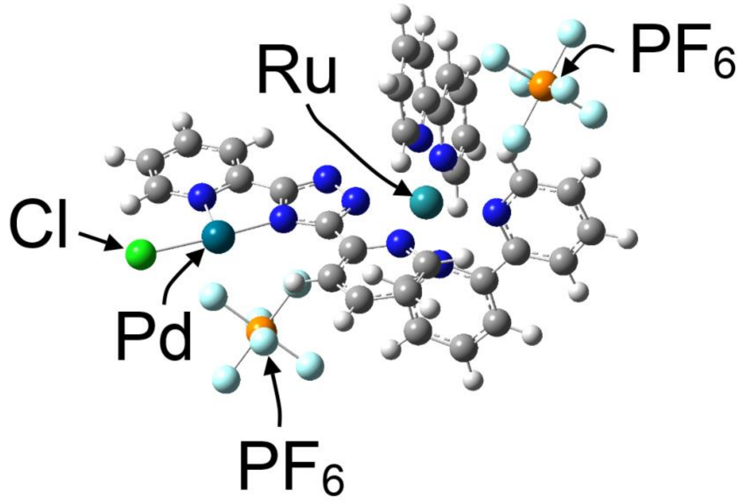

Figure S19. The optimised molecular structure (B3LYP/LanL2DZ) of $[Ru(bipy)_2(bpt)PdCl](PF_6)_2$ modelled in acetonitrile, showing the orientation used in the electron density difference maps.

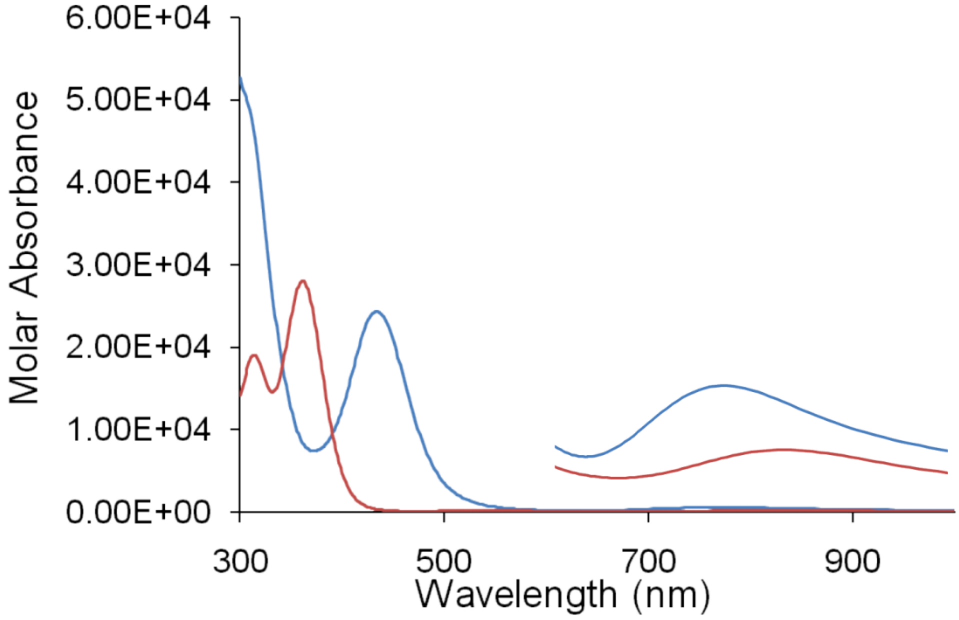

Figure S20. The simulated UV/vis spectrum of $[Ru(bipy)_2(bpt)PdCl](PF_6)_2$in acetonitrile, the blue spectrum was obtained using B3LYP/TD-DFT and the red spectrum was obtained using cam-B3LYP/TD-DFT, the inset is an expansion of the low energy region.

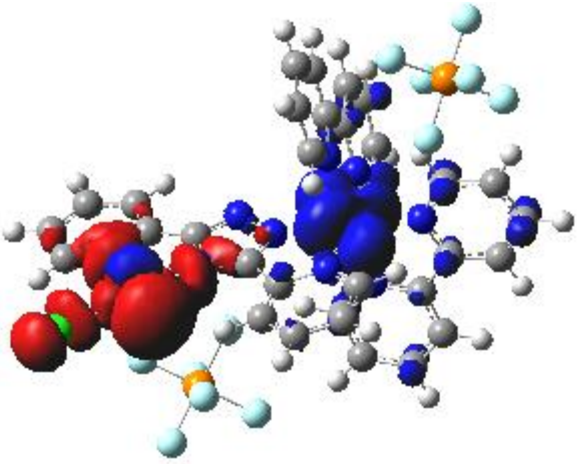

(a)

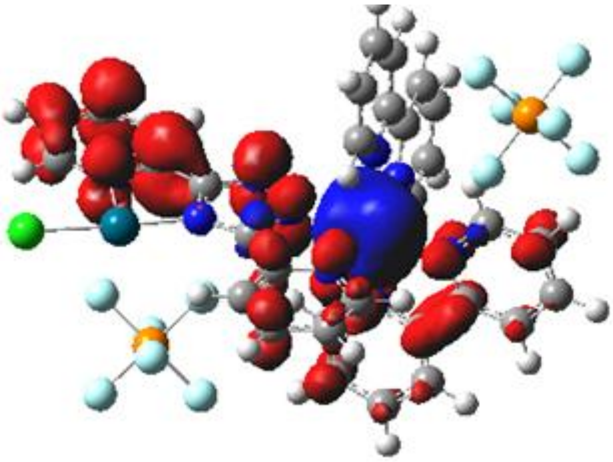

(b)

Figure S21. a) The electron density difference map illustrating the Ru to Pd charge-transfer nature of the third singlet excited state (vertical excitation energy 1.617 eV, corresponding to a photon wavelength of 766.7 nm); (b) the electron-density difference map for the 17th singlet excited state (2.866 eV, 432.6 nm) showing substantial Ru to bpt charge-transfer character. See text for explanation of colours. The molecular orientation is the same as that used in Figure S19.

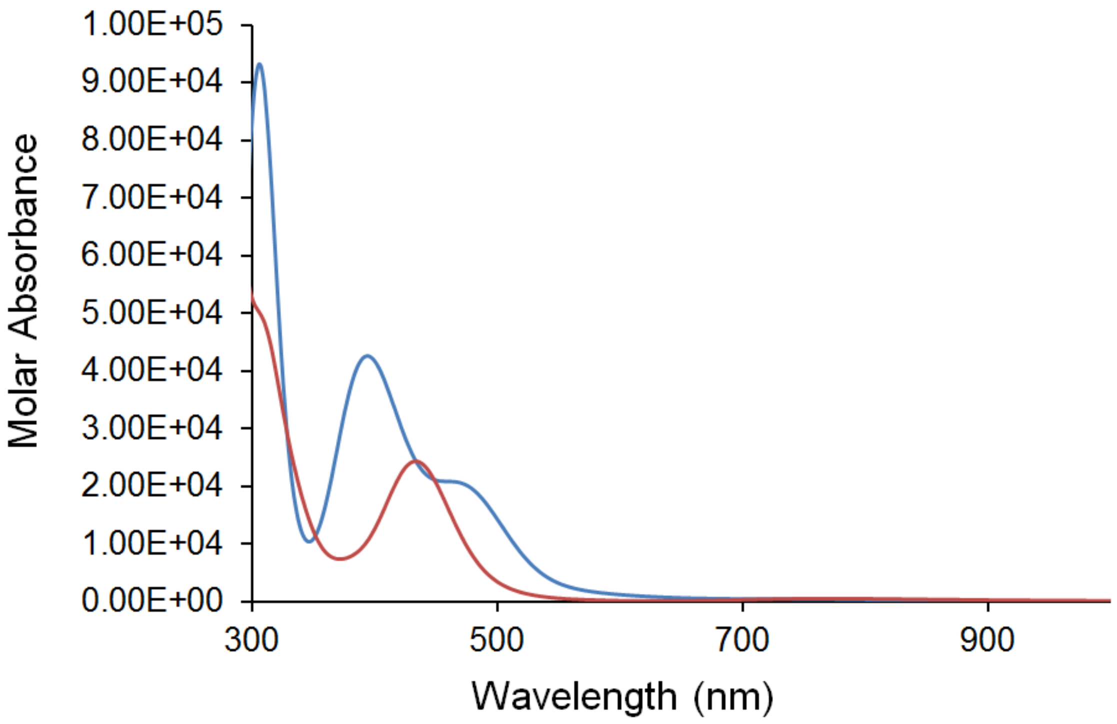

Figure S22. The simulated spectra of [Ru(dcmb)$_2$(bpt)PdCl](PF$_6$)$_2$ (blue) and [Ru(bipy)$_2$(bpt)PdCl](PF$_6$)$_2$ (red) in acetonitrile

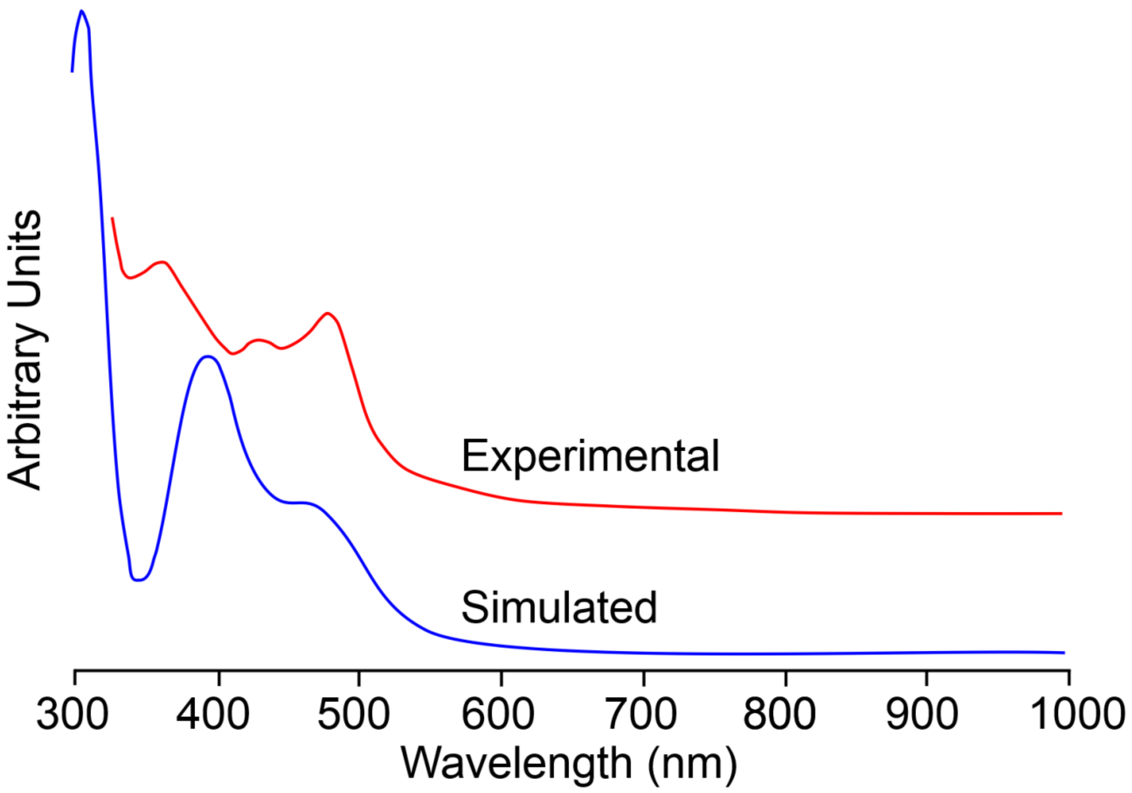

Figure S23. A comparison of the measured (red) spectrum [Ru(dceb)$_2$(bpt)PdCl] of and simulated (blue) UV/vis spectrum of [Ru(dcmb)$_2$(bpt)PdCl](PF$_6$)$_2$ in acetonitrile showing a similar asymmetry of the onset feature at approximately 480 nm.

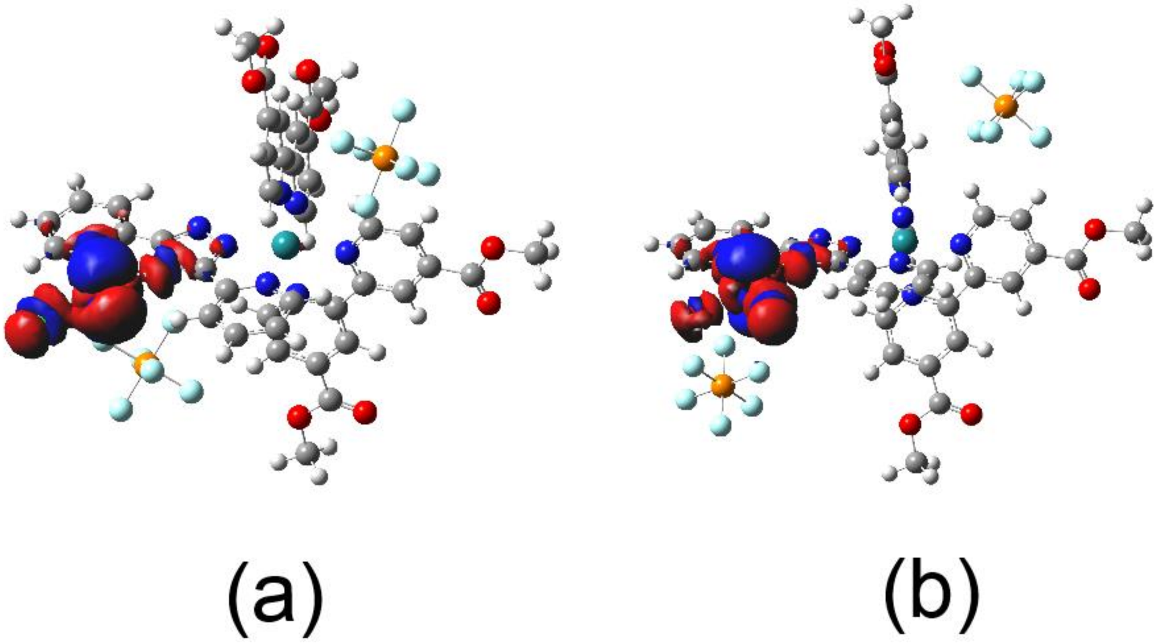

Figure S24. The electron density difference maps for the lowest energy triplet states of (a) $[Ru(dcmb)_2(bpt)PdCl](PF_6)_2$ in acetonitrile and (b) $[Ru(dcmb)_2(bpt)PdCl(H_2O)](PF_6)_2$ modelled in water showing the electron spin on the PdCl unit, these maps were obtained by subtracting the electron density of the ground singlet state at the triplet geometry from that of the optimised lowest energy triplet state (iso value of 0.0008)

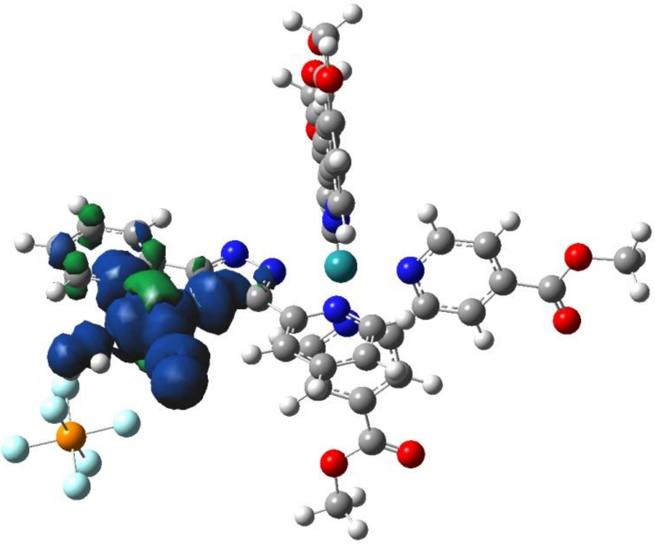

Figure S25 The spin density map for the singly reduced $[Ru(dmcb)_2(bpt)PdCl(H_2O)](PF_6)$ showing the unpaired spin on the Pd and its coordination sphere including the coordinated water.

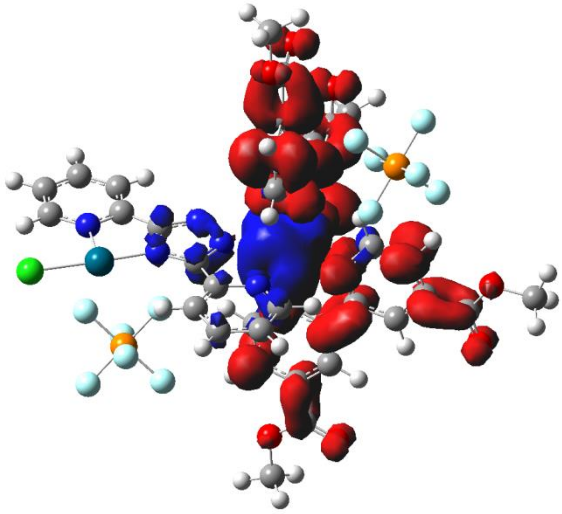

Figure S26. The electron density difference map for the lowest energy optically accessible singlet excited state (S12) of [Ru(dcmb)$_2$(bpt)PdCl](PF_6)$_2$ in acetonitrile showing the ruthenium-to-dcmb charge-transfer character of this state.

Transient absorption spectra

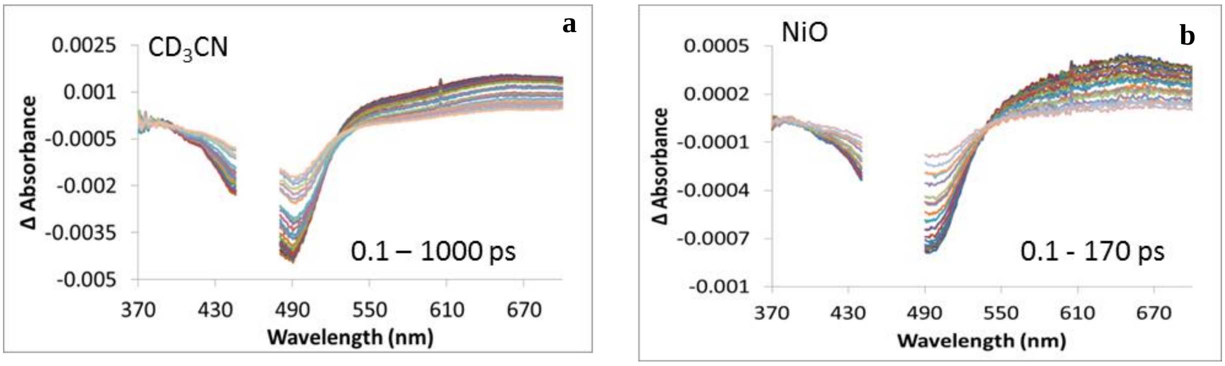

Figure S27. TA spectra for (a) compound **1** in CD_3CN and (b) **1**|NiO following excitation (λ = 470 nm).

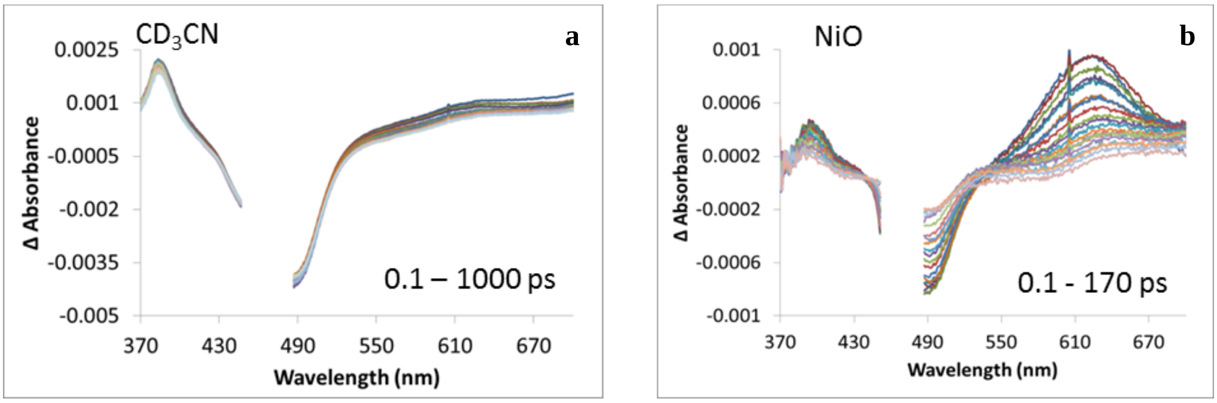

Figure S28. TA spectra for (a) compound **2** in CD_3CN and (b) **2**|NiO following excitation (λ = 470 nm).

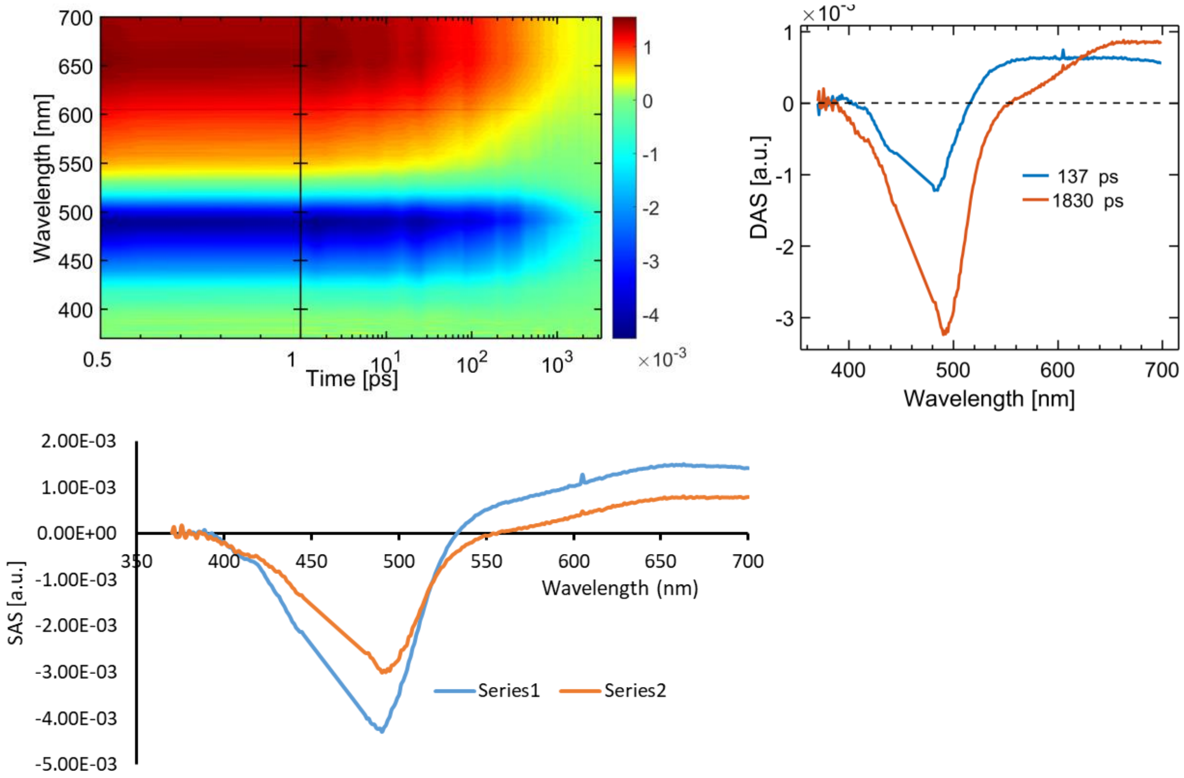

Figure S29. 3D TAS (top left) and DADS (top right) and SAS for compound **1** in CD_3CN following excitation (λ = 470 nm). SAS (Species-associated spectra) were constructed from DAS based on a model which assumes a consecutive reaction (no branching, no parallel, independent, co-existing species). The spectra do not change that dramatically with time, the longer-lived species have rather similar spectrum to the initial one, but the zero-crossing point shifts by ca. 20 nm, from 530 nm to 550 nm.

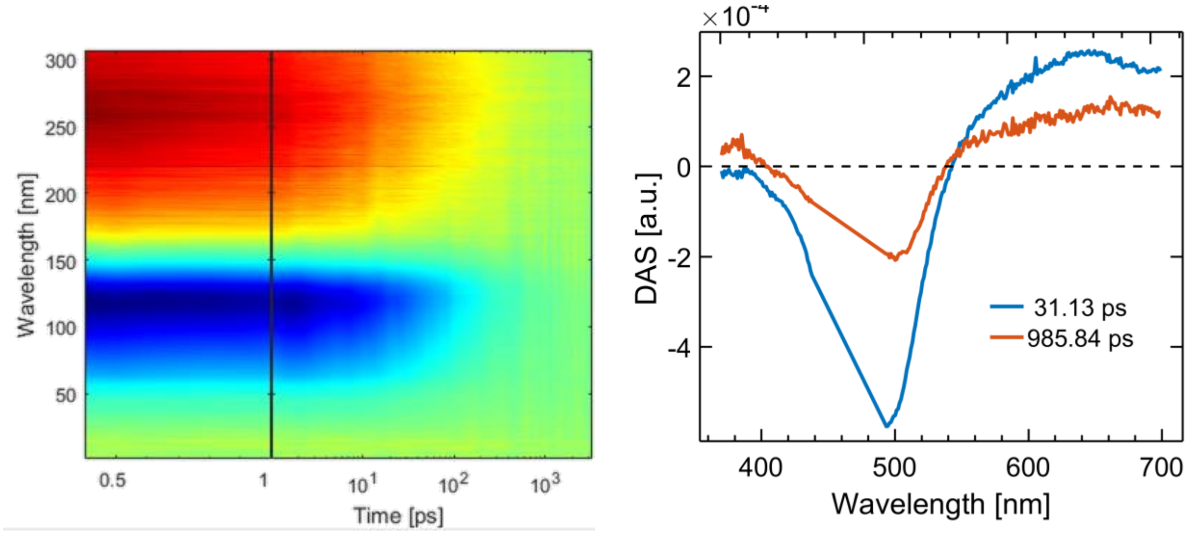

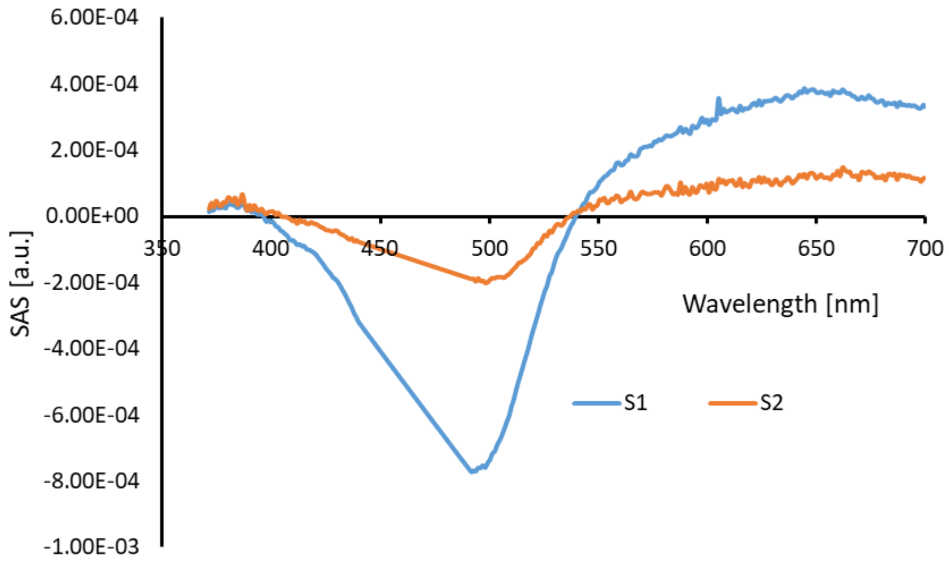

Figure S30. 3D TAS (top left), DADS (top right) and SAS (bottom) for compound **1**|NiO following excitation (λ = 470 nm).

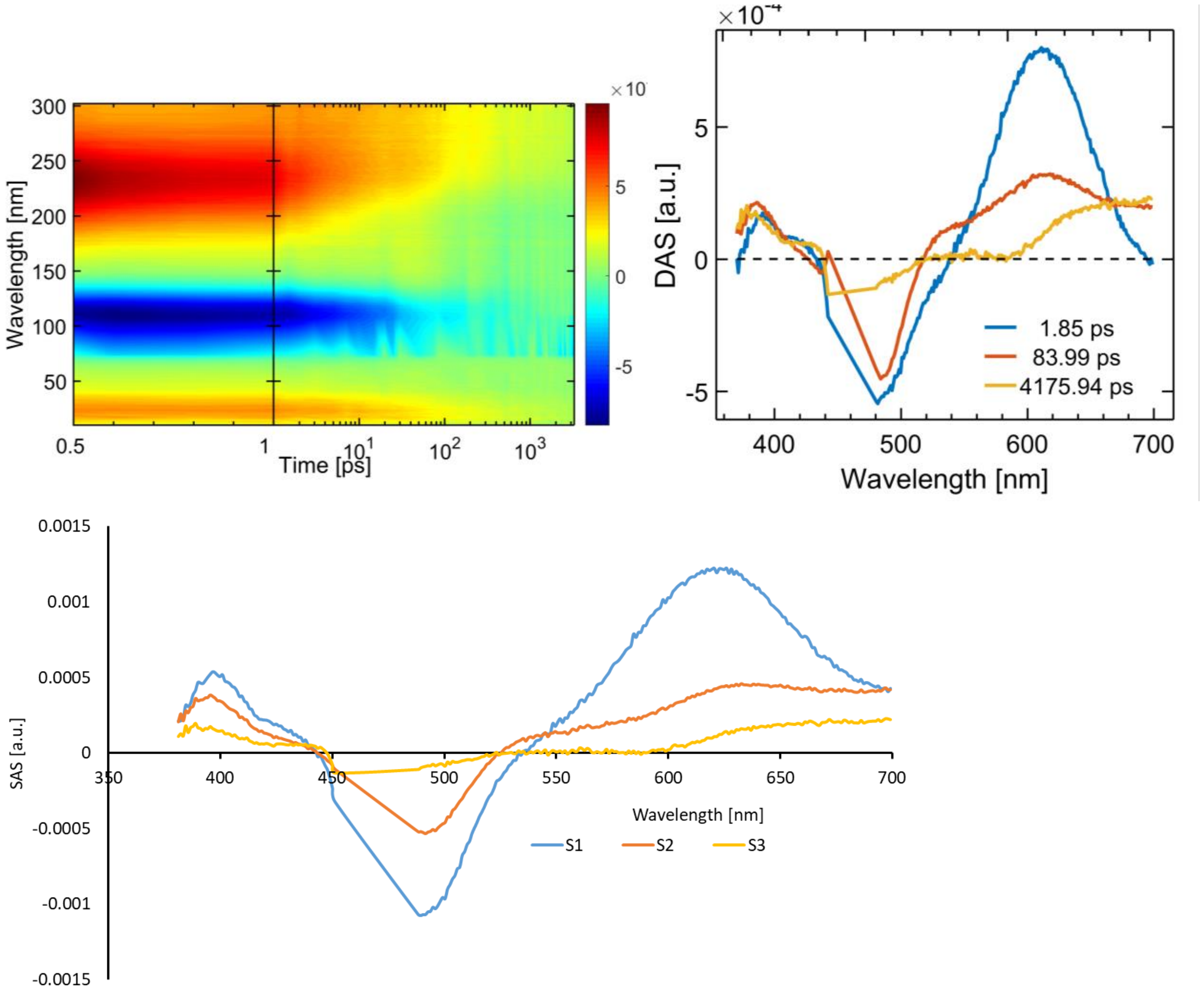

Figure S31. 3D TAS (top left), DADS (top right) and SAS (bottom) for compound **2**|NiO following excitation (λ = 470 nm). For 2 on NiO, all the three species show the TA band at about 380 nm you mentioned in the text, for S3, this maximum is shifted a bit further into the UV. The interesting feature to note is the change in the ground state bleach intensity when going to longer-lived species (also for Figure S30). The SAS are constructed assuming that there is no extra deactivating channels, so the SAS should represent the realistic intensity of the transient absorption spectra of the evolving species. So if there is no ground state repopulation until the very last state is populated, we would not expect to see much change in the ground state bleach between the states. This might mean that there is some branching during the excited state evolution, i.e. some additional deactivating route leading back to the ground state. A possible model is discussed in the main text.

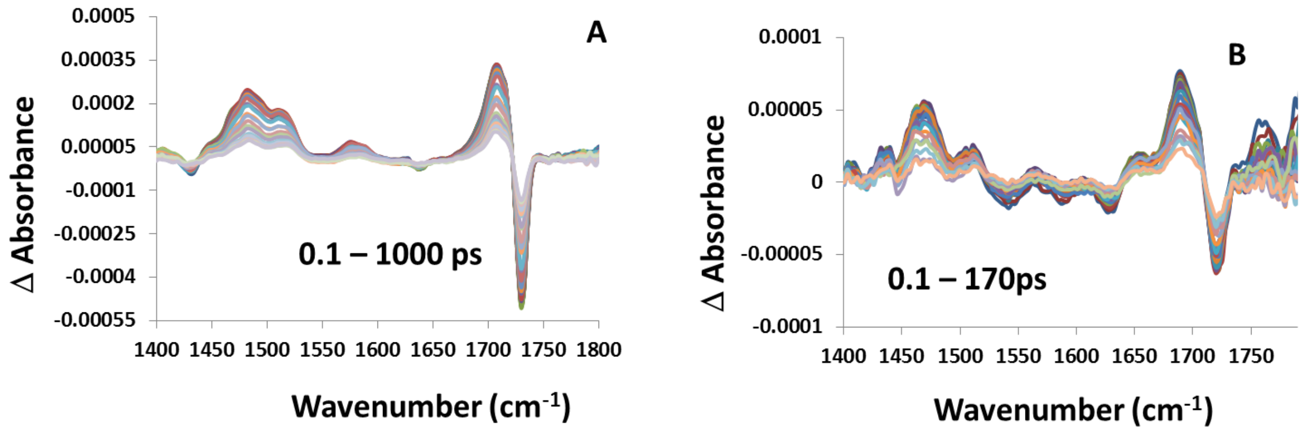

Figure S32, TRIR spectra of (A) compound **1** in CD3CN and (B) **1**|NiO following excitation at λ = 470 nm.

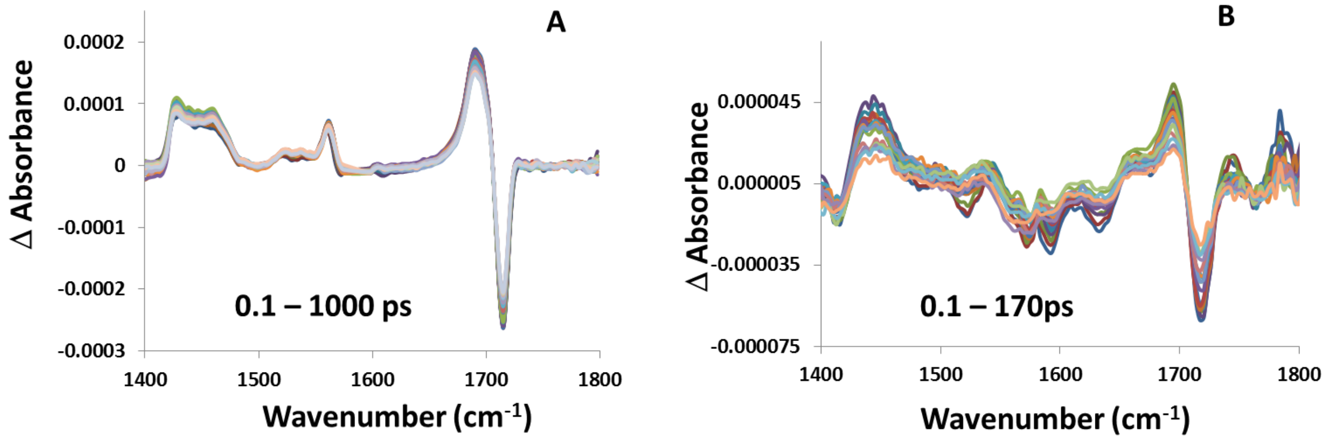

Figure S33, TRIR spectra of (A) compound **2** in CD_3CN and (B) **2**|NiO following excitation at λ = 470 nm.

Absorption spectra pre vs. post-catalysis

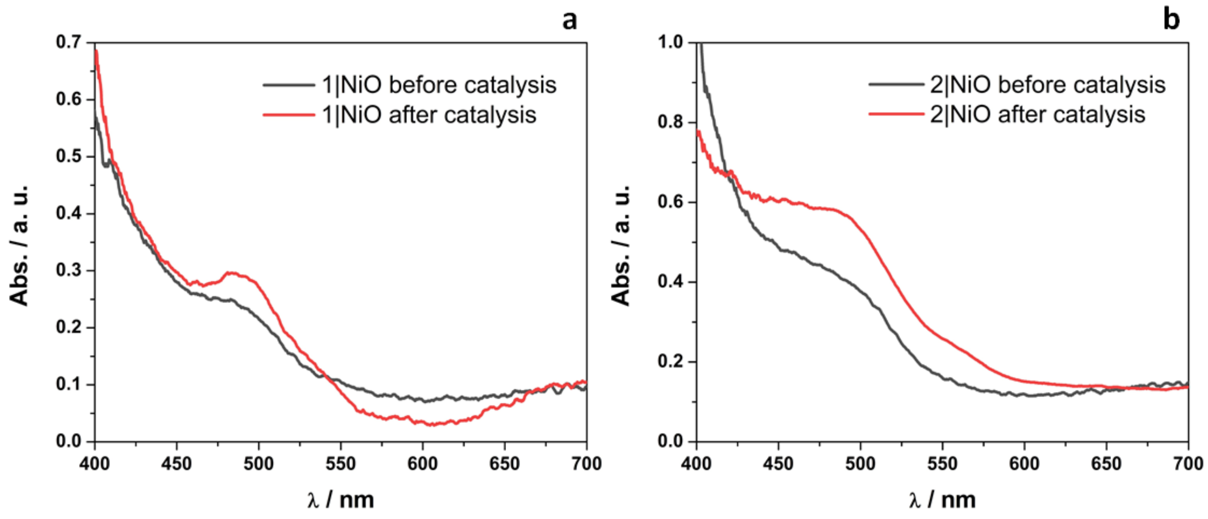

Figure S34. UV-vis spectra of (a) **1**|NiO and (b) **2**|NiO measured before and after photoelectrocatalysis at -0.4 V vs. Ag/AgCl (NiO background subtracted).

Control experiment $Ru(dcbpy)_3^{2+}$

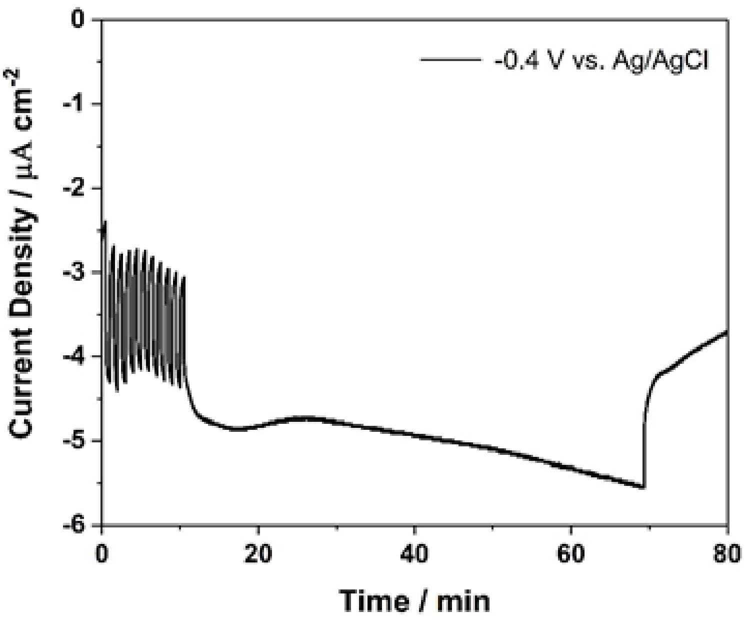

Figure S35. Chronoamperometry measurement of tris(2,2'-bipyridine-4,4'-dicarboxylic acid)ruthenium(II) dichloride | NiO in pH 3 aqueous electrolytes with 0.1 M potassium hydrogen phthalate. E_{appl} = – 0.4 V vs. Ag/AgCl reference electrode (3.0 M NaCl). Chopped light illumination was applied with 30 s intervals (10 cycles of dark current/photocurrent) followed by constant light illumination. Small photocurrent (1.5 µA) was recorded, but no hydrogen was detected when outlet gas from the PEC cell was analysed.

Mass spectrometry of **1**

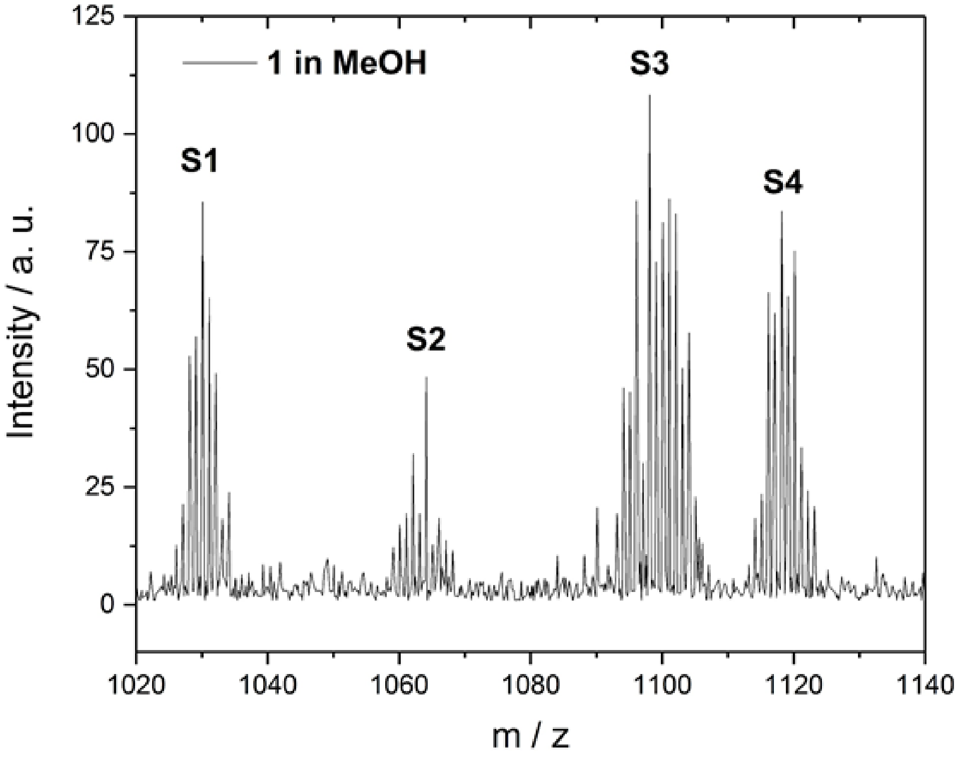

Figure S36. Mass Spectrometry of photocatalyst **1** in MeOH. Positive ion spectra. S1 - $[Ru(dceb)_2(bpt)Pd]^{2+}$ calcd. *m/z* = 1030, S2 - $[Ru(dceb)_2(bpt)Pd(CH_3OH)]^{2+}$ calcd. *m/z* = 1063, S3 - $[Ru(dceb)_2(bpt)Pd(CH_3OH)(H_2O)_2]^{2+}$ calcd. *m/z* = 1098, S4 - $[Ru(dceb)_2(bpt)Pd(CH_3OH)(H_2O)_3]^{2+}$ calcd. *m/z* = 1117 . Cl^- is substituted with MeOH in solution.

FTIR spectra of immobilised photocatalysts

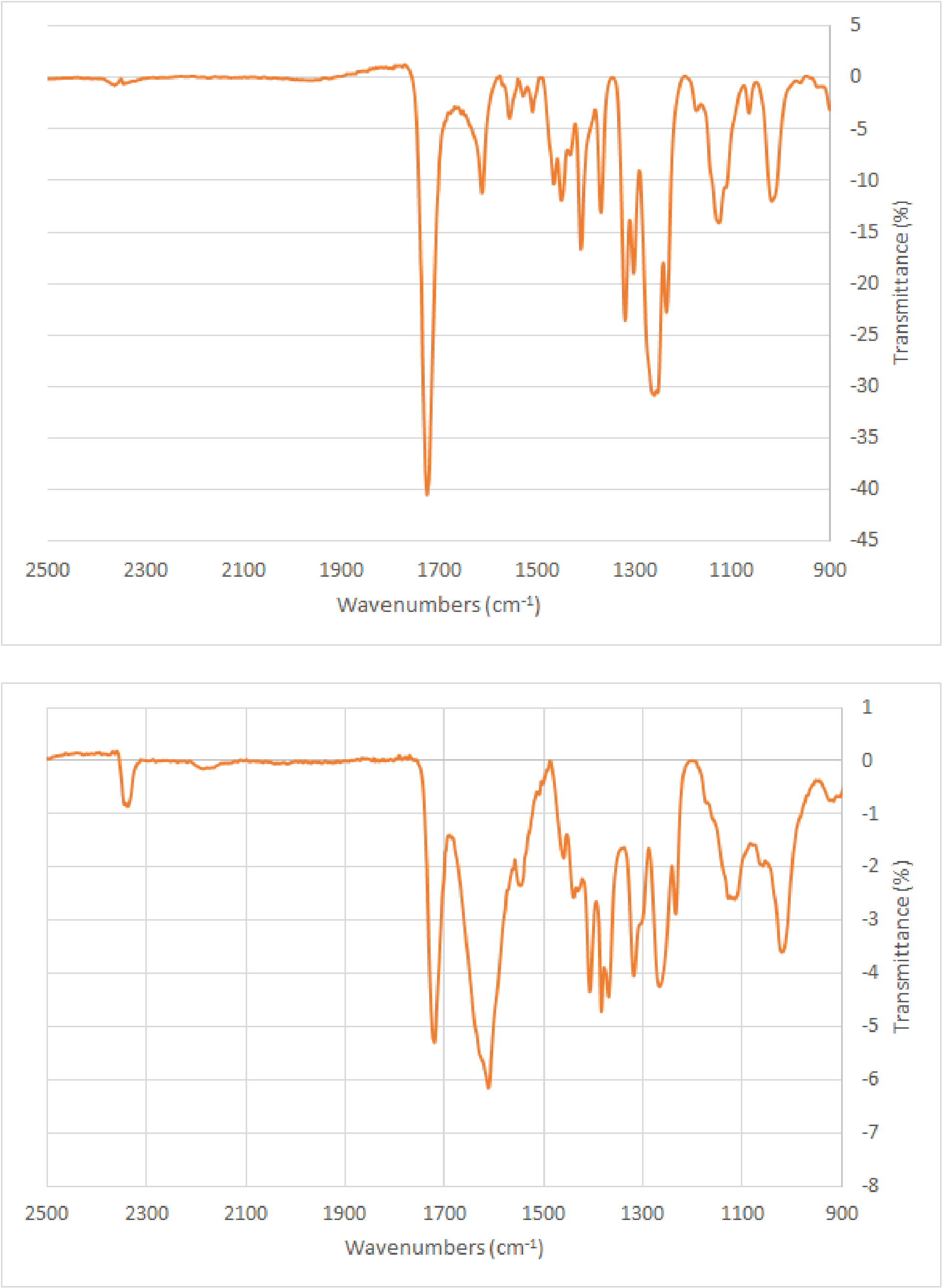

Figure S37. FTIR spectra of **1** (top) and **1**|NiO (bottom) in a KBr pressed disc.

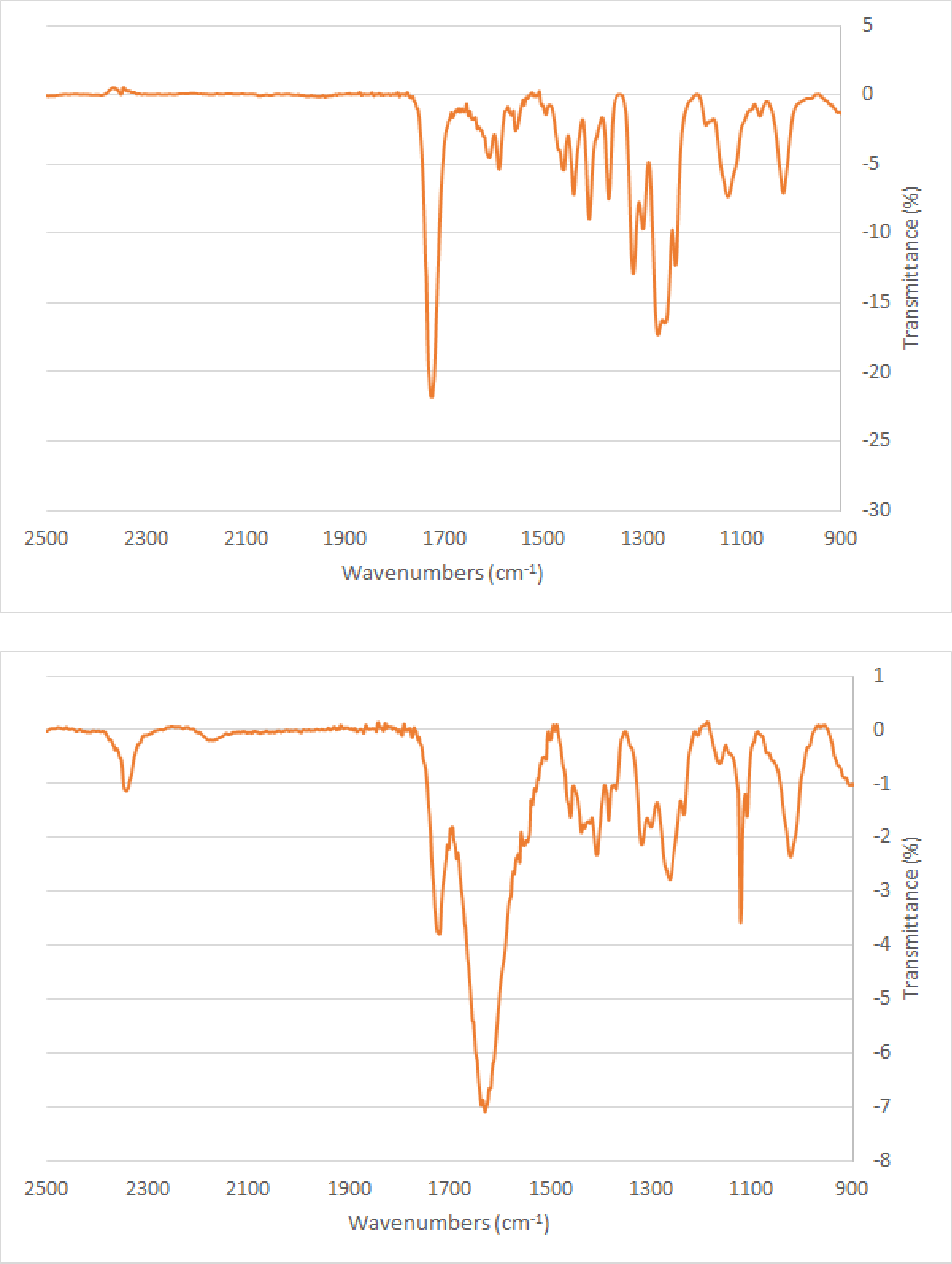

Figure S38. FTIR spectra of **2** (top) and **2**|NiO (bottom) in a KBr pressed disc.

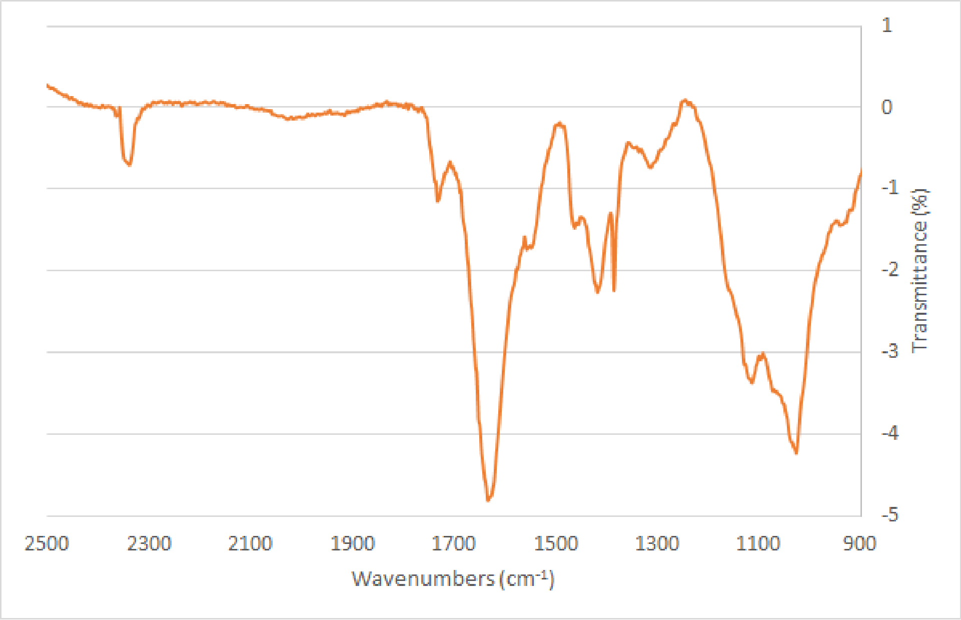

Figure S39. FTIR spectra of NiO in a KBr pressed disc.

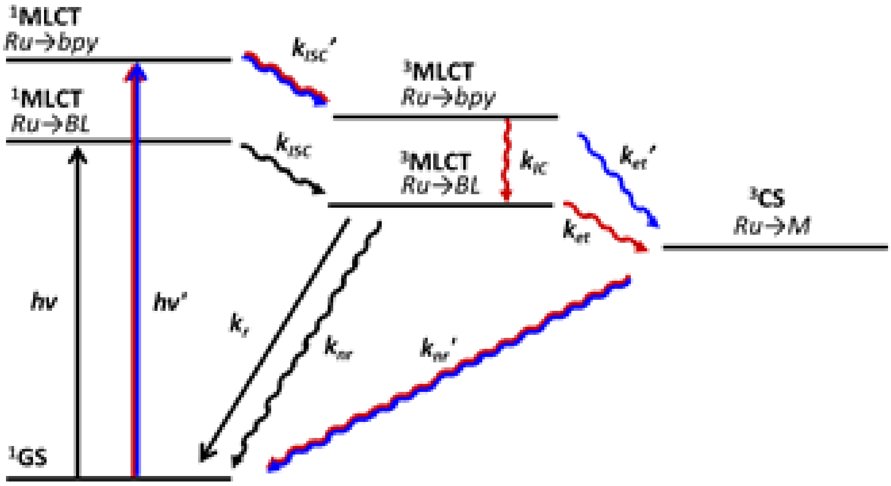

Figure S40. Proposed photoinduced pathways in **1** (red) and **2** (blue) based on the TA and TRIR experiments, DFT and TD-DFT calculations and information in references 21 and 25. solid arrows indicate radiative transitions and wavy arrows non-radiative transitions. BL = bridging ligand, bpy = bipyridyl, M = Pt or Pd, CS = charge-separated state.

References

1 G. Beamson and D. Briggs, *High Resolution XPS of Organic Polymers*, Wiley Interscience, 1992.

2 M. C. Biesinger, B. P. Payne, L. W. M. Lau, A. Gerson and R. S. C. Smart, *Surf. Interface Anal.*, 2009, **41**, 324–332.
